# Bacteriophage Therapy: Current Strategies and Future Perspectives

**DOI:** 10.1002/mco2.70645

**Published:** 2026-03-01

**Authors:** Zihe Zhou, Hanyu Fu, Mengzhe Li, Zhongyu Han, Zhenchao Wu, Huahao Fan, Ning Shen, Jiajia Zheng

**Affiliations:** ^1^ Department of Laboratory Medicine Peking University Third Hospital Beijing China; ^2^ Department of Pulmonary and Critical Care Medicine Peking University Third Hospital Beijing China; ^3^ State Key Laboratory of Green Biomanufacturing College of Life Science and Technology Beijing University of Chemical Technology Beijing China; ^4^ School of Life Sciences Tianjin University Tianjin China

**Keywords:** antimicrobial resistance, hospital‐acquired infections, phage–antibiotic synergy, phage‐based vaccines, phage therapy

## Abstract

Antimicrobial resistance represents a significant global health threat, demanding alternative treatments beyond traditional antibiotics. Phage therapy has resurged as a promising solution to address this challenge. This manuscript offers an in‐depth examination of phage applications in clinical settings, encompassing the treatment of multidrug‐resistant infections, prevention of hospital‐acquired infections, and development of phage‐based vaccines. Advanced strategies are explored, including phage–antibiotic synergy, biomaterial‐enhanced delivery systems to improve phage stability, and the rational design of engineered phages to expand host range and optimize lytic efficacy. Additionally, the application of genetic engineering to broaden phage host ranges and convert temperate phages into lytic variants is discussed. In hospital infection prevention, phages demonstrate substantial potential, such as eliminating bacterial biofilms on medical devices, disinfecting environmental surfaces, and controlling waterborne pathogens in hospital water systems. Furthermore, phages offer a versatile platform for vaccine development, facilitating efficient antigen display and nucleic acid delivery. Despite progress, challenges persist in pharmacokinetics, standardized production, and regulatory approval. This review synthesizes recent preclinical and clinical developments, emphasizing the transformative potential of phage‐based therapies while acknowledging the barriers to their clinical implementation.

## Graphical abstract

This manuscript systematically reviews the expanding scope of phage applications. It moves beyond traditional antibacterial use to explore their role in precision therapies against drug‐resistant infections, their synergy with antibiotics, and advanced biomaterial‐assisted delivery systems. The scope further extends to environmental decontamination (biofilm and water system control) and their innovative utility as versatile platforms for vaccine design, highlighting phages as multifaceted tools in medicine and biotechnology.

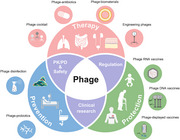

## 1 Introduction

Antimicrobial resistance (AMR) has become a critical global health threat, primarily manifesting as persistent bacterial infections in hospitalized patients—a challenge often overlooked by both patients and their families [1, 2]. In 2019, approximately 1.27 million deaths were directly attributed to drug‐resistant infections, with an additional 4.95 million deaths significantly linked to AMR [3]. Children under 5 years of age account for nearly 20% of all AMR‐related deaths, with major pathogens including *Klebsiella*, *Staphylococcus*, *Acinetobacter*, and *Escherichia* species [4, 5]. AMR also worsens the prognosis of patients with noninfectious diseases across all age groups, particularly the elderly. Currently, AMR ranks among the top three leading causes of death worldwide, following ischemic heart disease and stroke, and on par with chronic obstructive pulmonary disease [4].

In response, the World Health Organization (WHO) launched the Global Action Plan on Antimicrobial Resistance and published the first Bacterial Priority Pathogens List (BPPL) in 2017[6]. Since then, 14 new antibiotics have been approved by the United States Food and Drug Administration (US FDA) and the European Medicines Agency (EMA). However, with more than 80% of these being derivatives of existing antibiotics, concerns regarding the rapid development of resistance remain [7]. The antimicrobial agents currently in clinical development are insufficient to combat the ongoing rise and spread of drug‐resistant infections. As a result, WHO updated the BPPL in 2024 [8], utilizing a multicriteria decision analysis framework to incorporate new data and prioritize pathogens. Among these, carbapenem‐resistant Gram‐negative bacteria are of primary concern due to their complex resistance mechanisms, high capacity for horizontal gene transfer (HGT), and persistence in healthcare environments. Continued investment in the research and development of effective treatments and diagnostic tools for these pathogens is essential.

The pace of novel antibiotic development remains far behind the rapid rise of AMR. A key obstacle is the shrinking pool of viable drug targets, complicating the discovery of new therapeutic candidates. For instance, after screening 48,015 compounds for activity against Gram‐negative bacteria, the Global Antibiotic Research and Development Partnership was unable to identify any novel lead compounds suitable for further antibiotic development [9, 10]. Additionally, shifts in market dynamics between innovative drugs and generics have created an imbalance between investment risk and financial return, leading to a persistently stagnant market for new antibiotics. Global revenue from novel antibiotics peaked at $21 billion in 2001, but had steadily declined to $8 billion by 2021. In contrast, sales of generic antibiotics surged from $5 billion to $26 billion over the same two‐decade period [11].

In light of these challenges, nonantibiotic therapies, such as antimicrobial peptides and bacteriophages (phages), are gaining increasing attention. Phage therapy, which predates the advent of antibiotics, has been widely used in countries such as Russia and Georgia. A century later, phages have regained prominence, offering significant advantages, particularly in treating infections caused by multidrug‐resistant (MDR) bacteria that often evade conventional antibiotics. Over the past decade, global awareness of phage therapy has grown steadily, with several international phage consortia established and multiple clinical guidelines and expert consensus documents published [12]. This manuscript focuses on the diverse applications of phages in hospital settings, including the clinical treatment of MDR infections, the prevention and control of hospital‐acquired infections, and the development of phage‐based vaccines. By thoroughly evaluating existing preclinical and clinical data, this study outlines potential directions for the standardization of large‐scale phage production and the refinement of regulatory and scientific frameworks.

## 2 Strategies for Clinical Treatment with Phages

Given the limitations of antibiotics, phage therapy has re‐emerged as a promising alternative. The following section discusses strategies for the clinical application of phages.

### 2.1 Basic Biology, Pharmacokinetics, and Safety of Phage Therapy

#### 2.1.1 Biological Characteristics and Mechanisms of Action of Phages

The term “bacteriophage” originates from the Greek words meaning “bacteria eater,” referring to viruses that infect and kill bacteria. Like all viruses, phage particles consist of nucleic acid encased within a protein capsid [13]. Early classification based on morphological characteristics distinguishes tailed phages (including Myoviridae, Podoviridae, and Siphoviridae) from nontailed forms [14]. Phages are further categorized by the type of genomic nucleic acid into single‐stranded DNA, double‐stranded DNA (dsDNA), single‐stranded RNA (ssRNA), and double‐stranded RNA phages. Notably, over 95% of known phages possess linear dsDNA genomes [15]. Currently, phage classification is primarily based on genomic similarities, with phages organized into five families, 26 subfamilies, 363 genera, and 1320 species [16].

Another defining characteristic of phages is their inability to replicate outside the host, demonstrating strict host specificity [13]. Phages recognize and bind to specific receptors on the bacterial cell surface. Upon attachment, the phage injects its nucleic acid into the bacteria while the protein capsid remains outside. Some phages, like T5, employ a two‐step injection process in which a portion of the nucleic acid enters first, directing the synthesis of proteins necessary for the transfer of the remaining genome [17]. Lytic phages subsequently hijack the host's cellular machinery to replicate and assemble numerous progeny virions. The host cell eventually undergoes lysis, releasing newly formed phages, which can then infect adjacent bacteria, perpetuating the infection cycle. In contrast, lysogenic phages integrate into the bacterial genome as a prophage and replicate passively with the host. They enter the lytic cycle only when triggered by specific environmental stimuli [18]. Due to their ability to transfer antibiotic resistance genes (ARGs), virulence factors, and pathogenicity islands, temperate phages are generally deemed unsuitable for therapeutic applications in phage therapy [19].

#### 2.1.2 Pharmacokinetics and Pharmacodynamics of Phages

The absorption, distribution, metabolism, and excretion of phages is complex. Their self‐replication capacity influences not only the route of administration and dosage but also factors such as the presence or absence of target bacteria, the immunological status of the host, and the intrinsic biological properties of the phages [20]. This complexity suggests that conventional pharmacokinetics and pharmacodynamics (PK/PD) evaluation frameworks used for traditional drugs cannot be directly applied to phages, necessitating the development of novel assessment methodologies [21].

The absorption and distribution of phages in vivo are highly dependent on the route of administration, which serves as a key determinant of their PK profile. For localized infections, direct topical or regional delivery is an effective strategy to maximize phage concentrations at the infection site, facilitating rapid bacterial clearance. In the two‐part ELIMINATE Phase 2 trial for uncomplicated urinary tract infections (UTI), the phage cocktail LBP‐EC01 was administered intraurethrally. Following a dose of 2 × 10^12^ PFU, the mean peak phage concentration in urine reached 6.3 × 10^8^ PFU/mL [22]. This high‐concentration exposure aims to achieve a sufficient phage‐to‐bacterium ratio at the infection site, potentially inducing direct bacterial lysis through a “lysis from without” mechanism [23]. However, such approaches are limited to anatomically accessible sites. Intravenous administration is critical for achieving systemic distribution of phages to treat disseminated or deep‐tissue infections. Systemic exposure demonstrates dose dependency, as shown in the ELIMINATE trial: in Group A, receiving 1 × 10^10^ PFU intravenously, the mean peak plasma concentration (*C*_max_) was 4.0 × 10^3^ PFU/mL, while in Group C, receiving 1 × 10^11^ PFU via continuous infusion, *C*_max_ reached 8.0 × 10^5^ PFU/mL [22]. Phage titers in plasma were quantified using the standard double‐layer agar plaque assay [22, 24]. Oral administration offers the best patient adherence and is primarily suited for gastrointestinal infections, where high local intestinal concentrations can be achieved, although systemic bioavailability is typically low. In a shrimp model infected with *Vibrio parahaemolyticus*, orally or intragastrically administered phages rapidly distributed to the intestine, hepatopancreas, and hemolymph, with the highest titers observed in intestinal tissues despite notable intertissue concentration variations [25, 26]. Similarly, in the ELIMINATE trial, no phages were detected in the plasma of two orally dosed patients, while fecal phage levels reached up to 3.1 × 10^8^ PFU/g, indicating limited intestinal absorption [22]. Coadministration with antacids or microencapsulation techniques are often used to enhance phage stability and absorption [27].

The metabolic and excretory pathways of phages in vivo differ fundamentally from those of small‐molecule drugs. Unlike conventional pharmaceuticals, phages are not metabolized by hepatic cytochrome P450 enzymes and are not primarily eliminated via renal excretion due to their large particle size, which exceeds the glomerular filtration threshold [28]. Instead, phage clearance is mainly mediated by the host immune system. As foreign particles, phages can be opsonized by the complement system or directly recognized and internalized by phagocytes [29]. This rapid, nonspecific innate immune clearance is the primary mechanism for phage removal from circulation, explaining why intravenously administered phages typically exhibit short plasma half‐lives ranging from minutes to hours in animal models [20, 30]. This clearance persists even in individuals who have not developed specific antibodies [31]. Prolonged or repeated exposure to phages can induce adaptive immune responses, resulting in the production of neutralizing antibodies. These antibodies primarily target phage tail structures, blocking adsorption to bacteria and leading to neutralization [32, 33]. Phage‐neutralizing antibodies can be detected directly by ELISA [34] or assessed indirectly through phage neutralization assays [35]. However, antibody levels do not always correlate with clinical outcomes. While some patients have been successfully treated despite antibody development, one immunocompetent patient experienced treatment failure after 2 months of intravenous phage therapy due to the formation of neutralizing antibodies [36]. Consequently, adjuvant strategies, such as phage cocktails, rotation of different phages, or engineering approaches to reduce immunogenicity, should be considered, along with regular monitoring during treatment [37], to mitigate or delay the adverse effects of neutralizing antibodies.

The multiplicity of infection (MOI) is a critical PD parameter influencing the bactericidal efficacy of phages. Achieving a high local MOI (≥1) theoretically ensures that each bacterial cell is adsorbed and lysed by phages. However, in systemic infections, maintaining a high MOI across all infection sites is challenging due to phage dilution throughout the body and rapid immune system clearance. Under such conditions, the therapeutic efficacy of phages depends on their amplification at the infection site, leading to dynamic changes in MOI. Therefore, treatment outcomes rely not only on the initial dose administered but also on the rate and extent of phage amplification within the target bacterial population. Mathematical models, such as PHORCE, are being developed to quantify phage amplification rates, offering a novel approach to understanding in vivo PD [38].

Despite advancing understanding, research on the PK/PD of phages continues to face significant challenges that impede clinical translation. Existing PK data are derived from a variety of phages, animal models, infection types, and dosing regimens, leading to considerable heterogeneity. The absence of standardized detection methods and data‐analysis models complicates efforts to draw generalizable conclusions [39]. The EMA has begun requiring nonclinical studies of phages (including in vitro and animal experiments) to provide PK/PD information [12]. Cairns et al. conducted an in vitro PK/PD study of *Campylobacter jejuni* GIC8 and phage CP8, highlighting the potential of quantitative pharmacological models to optimize phage dosing and administration strategies. However, further exploration is needed for clinical translation [40]. Additionally, interspecies differences in immune systems pose challenges in extrapolating animal PK/PD data to humans. For instance, the intensity and timing of neutralizing antibody production in humans may differ from those in rodents [41].

#### 2.1.3 Safety Profile of Phage Therapy

Available clinical trial data indicate that adverse events associated with phage therapy are generally mild to moderate in severity, with serious events being rare [42]. However, the biological nature of phages introduces specific risks. A primary concern is the potential for phages to induce neutralizing antibodies, a phenomenon particularly evident in animal models [43]. Although individual responses vary, such immune reactions may compromise the efficacy of repeated treatments and require close monitoring through therapeutic phage tracking [44]. Additionally, the rapid lysis of Gram‐negative bacteria by phages can release endotoxins, potentially triggering transient inflammatory reactions such as chills, fever, or tachycardia, as observed in some high‐dose intravenous administration cases [42]. Therefore, phage production must comply with good manufacturing practice (GMP) standards to control endotoxin levels and prevent impurities. While lytic phages used therapeutically are unlikely to transfer ARGs, screening for potential virulence or resistance genes remains a critical aspect of modern formulation development [45]. Following intravenous administration, phages are widely distributed across various tissues and organs, including the central nervous system. No studies have reported neurological dysfunction resulting from therapeutically administered phages [46], and some research even confirms the natural presence of phage particles in the nervous system [47]. Nevertheless, the long‐term neurological safety of phage therapy warrants further investigation in future clinical studies [48].

In summary, the safety profile of phage therapy is considered manageable. The primary risks are transient effects linked to immune responses and bacterial lysis, rather than the toxicity commonly associated with conventional drugs. With the standardization of manufacturing processes and enhanced clinical monitoring, the safety of phage therapy is expected to be further improved.

### 2.2 Single Phage or Phage Cocktail Therapy

In current preclinical and some clinical studies, phage therapy primarily utilizes two formulation types: natural single phages and phage cocktails. Single phages are preferred for their target specificity, facilitating research into their mechanisms of action and safety evaluations. However, their application is limited by the heterogeneity of target bacterial strains and the potential for phage resistance development. In contrast, phage cocktails combine multiple distinct phages, offering broader strain coverage, reducing the risk of resistance development, and demonstrating superior therapeutic efficacy in polymicrobial infections [49, 50]. Additionally, the design of phage cocktails can be optimized through modern techniques such as genomic surveillance and high‐throughput screening, enabling precise matching of target strains and enhancing treatment specificity and efficacy [50, 51]. This section reviews the research progress of natural single phages and phage cocktails, analyzes their application effects and mechanisms in infections caused by various pathogens, and discusses the advantages and limitations of both approaches in clinical settings.

#### 2.2.1 Current Main Implementation Approaches of Phage Therapy

The use of preselected broad‐spectrum phages involves employing a single or fixed combination of phages that are effective against multiple strains of specific pathogenic bacteria. The core principle of this method is to utilize broad‐spectrum phages directly in clinical applications, thus eliminating the need for strain‐specific screening in patients and enabling a rapid, immediate response. The advantages of this approach include a fast response time and relatively low cost [52]. Preselected phages, having undergone thorough identification and validation, can be produced and stockpiled on a large scale. This allows for prompt deployment in emergency or empirical treatment scenarios, while also enhancing quality control and streamlining regulatory approval processes. However, the limitations of this approach are notable. The lytic spectrum of broad‐spectrum phages may not cover all clinical strains, potentially leading to treatment failure. Furthermore, overreliance on a limited number of broad‐spectrum phages may accelerate bacterial resistance, undermine long‐term efficacy, and pose public health risks. Additionally, this approach is poorly adaptable to emerging or rare strains [53]. Consequently, while suitable for rapid responses to common infections due to its cost‐effectiveness and ease of production, it requires constant vigilance against coverage gaps and resistance risks. Future efforts should focus on expanding phage library diversity, optimizing combinations, and enhancing resistance surveillance [54].

The patient strain isolation and matching (“personalized”) approach involves isolating pathogenic bacteria from the patient and screening for phages capable of lysing the specific strain. This method's core principle is achieving precise antibacterial action tailored to the individual bacterial strain. It offers high specificity and potentially superior efficacy, significantly reducing the risk of mismatched lytic spectra. This approach is particularly beneficial for treating rare or MDR bacterial infections and provides flexible, individualized treatment solutions [55]. However, its primary challenges include time‐consuming and complex procedures. Strain isolation, screening, and formulation preparation can take days to weeks, making it difficult to meet urgent clinical needs. Additionally, the high technical barriers and costly processes limit widespread adoption, and the approach relies heavily on large‐scale, diverse phage library resources [56]. Despite these challenges, the personalized approach has shown promise in treating complex infections, such as refractory pulmonary or osteoarticular infections. To achieve clinical translation, rapid screening platforms, standardized production processes, and appropriate regulatory frameworks must be established [57, 58].

#### 2.2.2 Design Strategy and Scientific Rationale for Phage Cocktails

The design objectives of phage cocktails primarily focus on three key aspects: broadening the lytic spectrum, enhancing bactericidal efficacy, and preventing or delaying resistance development. First, expanding the lytic spectrum aims to ensure that the cocktail can target multiple serotypes or genotypes of pathogenic bacteria, thus enhancing the treatment's applicability. For example, the design of phage cocktails targeting plant pathogens emphasizes broadening the host range to limit bacterial resistance development while preserving phage lytic activity [59]. Second, enhancing bactericidal efficacy is achieved through the synergistic interactions of multiple phages, allowing for faster and more thorough bacterial clearance. Single phages often lead to rapid resistance development by targeting a single receptor, whereas multiphage cocktails can target different receptors, significantly improving treatment efficiency [60, 61]. Third, preventing or delaying resistance emergence is a core strategy in phage cocktail design. By utilizing different phages to target multiple receptors on the bacterial surface, the likelihood of bacterial escape via single‐receptor mutations is minimized, thereby prolonging therapeutic efficacy [60]. Phage cocktails targeting Salmonella, for instance, have demonstrated significant delays in resistance development and enhanced antibacterial effects by combining phages that recognize distinct receptors [62]. Therefore, when designing phage cocktails, it is crucial to consider the target bacterial species and their genetic diversity, combining multiple phages to cover different serotypes, enhance synergistic bactericidal activity, and effectively prevent resistance. These principles form the foundation of efficient treatment design.

A primary mechanism by which bacteria develop resistance to phages is by evading infection through mutation or loss of surface receptors critical for phage adsorption. If all phages in a cocktail rely on a single receptor, bacteria can evade the entire phage attack with just one mutation of that receptor, leading to treatment failure [60]. Hence, receptor‐targeting diversity is a critical principle in the design of phage cocktails. Specifically, selecting phage combinations that target different molecular receptors—such as flagella, pili, lipopolysaccharides (LPS), and outer membrane proteins (e.g., OmpA, ComEA)—can establish a multilayered protective barrier [63, 64]. For example, a four‐phage cocktail targeting *Salmonella* was designed to bind different components of LPS and outer membrane proteins, significantly delaying the emergence of resistant strains. Moreover, the resistant variants exhibited increased susceptibility to antibiotics and reduced virulence [60]. This diversity of receptors enhances therapeutic robustness and extends the effective lifespan of the cocktail, representing a core strategy for achieving sustained clinical efficacy [61, 65].

In terms of strategic application, the design should account for the diversity of surface receptors among target bacteria, screening and combining phages that can effectively recognize various receptors. Recent studies have employed genome sequencing and structural biology techniques to analyze the interactions between phage receptor‐binding proteins (RBPs) and bacterial receptors, thereby guiding the rational composition of phage cocktails [66, 67]. Furthermore, some studies have enhanced the host range and specificity of phage cocktails by constructing chimeric tail fiber proteins tailored to target distinct receptors [68]. In summary, receptor‐targeting diversity not only prevents the rapid spread of single‐resistance mutations but also promotes synergistic antibacterial effects, significantly enhancing the clinical potential of phage cocktails.

#### 2.2.3 Comparison and Selection Considerations Between Single Phage and Phage Cocktail

A single phage typically targets specific bacterial strains or closely related bacterial groups, demonstrating high lytic efficiency. Due to its narrow lytic spectrum, a single phage exhibits a high degree of specificity, providing precise bactericidal effects against particular pathogens. For instance, ssRNA phages, such as MS2, with their compact structure and strong infection specificity, efficiently infect and lyse certain Gram‐negative bacteria [68, 69, 70]. However, this specificity limits its broader application, as the therapeutic efficacy of a single phage may be compromised when faced with bacterial strain diversity and heterogeneity. In contrast, phage cocktails combine multiple phages with varied lytic spectra, extending coverage to a broader range of bacterial populations and improving the ability to address bacterial heterogeneity. The cocktail approach reduces the likelihood of bacterial resistance by targeting multiple bacterial receptors, thus enhancing overall therapeutic efficacy. Studies on the crop pathogen Erwinia amylovora have demonstrated that phage cocktails containing several phages exhibit synergistic inhibitory effects against different bacterial strains [71]. Similarly, multiphage combinations targeting *Salmonella enterica* display high lytic activity across diverse strains [72]. Furthermore, phages within a cocktail may target distinct receptors or bacterial surface structures, further broadening the lytic spectrum and improving treatment comprehensiveness. From the perspectives of efficacy and spectrum, single phages are best suited for treating known sensitive strains, whereas phage cocktails are more appropriate for complex, diverse bacterial infections, offering distinct advantages in scenarios involving significant microbial heterogeneity or a high risk of drug resistance [59, 62].

A major challenge in single‐phage therapy is the increased risk of bacterial escape through receptor variation or other resistance mechanisms. Studies have shown that bacteria such as *Pseudomonas* protegens can modify their surface glycosylases via genetic mutations (e.g., amino acid substitutions in AlgC and YkcC), reducing phage receptor accessibility and evading infection to maintain a survival advantage [72]. This highlights that single‐phage therapy is vulnerable to rapid bacterial evolution, which can quickly lead to resistance. In contrast, rationally designed phage cocktails, particularly those containing multiple phages targeting different bacterial receptors, significantly reduce the rate of bacterial resistance development. Multitarget strategies require bacteria to undergo several simultaneous mutations to evade all phages, greatly diminishing the likelihood of resistance due to compounded evolutionary pressure [73]. For instance, a phage cocktail targeting Pseudomonas aeruginosa (*P. aeruginosa*) effectively prevented the emergence of resistant strains by combining phages that target different receptors [73]. Additionally, the synergistic effects among multiple phages can enhance overall efficacy while suppressing bacterial resistance evolution. In summary, while single‐phage therapy carries a substantial risk of resistance, phage cocktails utilize multitarget and multimechanism strategies to reduce resistance development, thereby improving treatment durability and stability.

From the perspectives of development and regulation, single phage preparations are relatively straightforward in terms of characterization, production, and quality control due to their homogeneous composition. Assessing genomic integrity, purity, and titer is more direct for single phages, and their production processes are well established, facilitating large‐scale manufacturing and standardized management [74]. In clinical applications, evaluating and monitoring the safety and efficacy of single phage preparations is simpler. In contrast, the development of phage cocktails is more complex. Optimizing the ratios of different phage components is necessary to ensure stability and overall potency. Potential interference or antagonistic effects among phages require comprehensive in vitro and in vivo efficacy evaluations to determine the most effective combination [61, 75]. Additionally, phage cocktails demand more stringent safety testing, addressing the interactions between components and the potential risks of immune responses. During regulatory approval, phage cocktails, due to their complex composition, require more extensive clinical data, leading to a more burdensome approval process compared with single phages [76]. Consequently, single phages demonstrate greater efficiency in both development and regulatory processes, while cocktails, despite their enhanced efficacy, involve higher development costs and regulatory challenges due to their complexity.

Single‐phage therapy is particularly suitable for infections involving known susceptible bacterial strains, offering significant advantages in precision medicine and on‐demand treatment. For instance, when clinical testing identifies the pathogenic bacteria and confirms their sensitivity to a specific phage, single‐phage therapy serves as an effective targeted treatment option [77, 78]. Single phages are also commonly used for monitoring and rapid intervention in specific environments or against particular pathogens. In contrast, phage cocktails are more appropriate for empirical therapy, especially when dealing with highly heterogeneous pathogen populations. Their broad lytic spectrum effectively targets multiple bacterial strains, reducing the risk of treatment failure due to resistant strains [62, 79]. Cocktails are often employed as the preferred strategy for resistance prevention, with extensive applications in agriculture, food safety, and other industries [59, 80]. In environments with mixed or complex infections, phage cocktails provide broader antimicrobial coverage. Thus, single phages are ideal for precise, highly targeted treatment, while cocktails are better suited for complex, diverse infections and therapeutic needs aimed at preventing resistance.

Natural single phages and phage cocktails, the two primary modalities in contemporary phage therapy, each offer distinct advantages and are suited to different applications, demonstrating a complementary and coexisting relationship. Future development must strike a balance between these two approaches. On one hand, efforts should be focused on establishing extensive, well‐characterized phage libraries, combined with high‐throughput screening and genomics technologies, to enable rapid and precise matching of phages to specific pathogens. On the other hand, intelligent cocktail design platforms will be essential, leveraging artificial intelligence and machine learning to optimize formulation combinations and improve the accuracy and personalization of efficacy predictions. Additionally, advancements in rapid diagnostic technologies will facilitate the quick identification of pathogenic bacteria at clinical sites, enabling the prompt selection or adjustment of preprepared phage cocktails. This approach integrates “broad‐spectrum prevention” with “personalized treatment,” offering flexibility in phage therapy applications. By developing a more comprehensive and efficient antibacterial treatment system, these innovations hold the potential to revolutionize the prevention and control of MDR infections, marking a new era in the antimicrobial field.

### 2.3 Phage–Antibiotic Synergy

Although numerous case reports have documented pathogen clearance or clinical improvement following personalized phage therapy, such treatments remain predominantly compassionate use interventions [13]. According to the US FDA, compassionate use refers to providing an investigational drug outside of clinical trials for patients with serious or life‐threatening conditions who lack approved treatment options or cannot participate in relevant trials [81]. As therapeutic agents, phage preparations have not yet received widespread regulatory recognition regarding their safety and efficacy. Moreover, the absence of comprehensive phage‐specific legislation within modern pharmaceutical regulations further hinders their formal approval. Consequently, combining phages with standard antibiotic therapy appears to be a more clinically feasible approach at present.

In vitro studies have demonstrated that combining phages with β‐lactams, tetracyclines, or quinolones significantly reduces the minimum inhibitory concentrations (MICs) of these antibiotics. Notably, the MIC of fluoroquinolones can decrease from 2 µg/mL to as low as 0.06 µg/mL [82, 83]. In vivo research further supports that phage–antibiotic combinations substantially improve survival in infection models. For example, Zhao et al. [49] treated murine intra‐abdominal infections with either a phage cocktail alone or a combination of phages and antibiotics. The combination therapy reduced *Klebsiella pneumoniae* (*K. pneumoniae*) loads by a factor of ten more effectively than the phage cocktail alone. Phage–antibiotic interactions depend heavily on the type of antibiotics used. Several clinical reports reinforce the efficacy of personalized phage therapy combined with antibiotics. Stellfox et al. [84] described a case of recurrent *Enterococcus faecium* (*E. faecium*) bloodstream infection, where a phage cocktail, combined with vancomycin and daptomycin, reduced intestinal colonization of vancomycin‐resistant *E. faecium*, prevented recurrent bacteremia, and improved clinical symptoms. Chan et al. [85] also reported the successful treatment of a chronic *P. aeruginosa* infection using phage OMKO1 in combination with ceftazidime, with no recurrence observed. In another case involving a fracture‐related infection caused by pan‐drug‐resistant *K. pneumoniae* [86], the patient received an experimental regimen consisting of phage vB_KpnM_M1 from the Eliava Institute, along with a phage variant isolated after coevolution with the pathogen, combined with ceftazidime‐avibactam. After a brief treatment course, the pathogen was no longer detected in the wound, and the patient's overall condition improved. Phage therapy in this case was delayed for 2 years following ethics approval due to disagreements among the treating physicians and was only initiated after multiple conventional antibiotic regimens had failed. Building on this experience, the team later used phage cocktail BFC1[87], which includes one *Staphylococcus aureus* (*S. aureus*) phage and two *P. aeruginosa* phages, combined with several antibiotics to successfully control bloodstream infection and liver abscess in a pediatric patient following liver transplantation. This outcome suggests that prolonged intravenous phage administration can be safely tolerated, even in immunocompromised children. In summary, synergistic effects between phages and antibiotics have been demonstrated in multiple studies. Phage–antibiotic combination therapy shows efficacy against both Gram‐positive and Gram‐negative bacteria and exhibits a favorable safety profile, even in immunodeficient patients. These findings strongly support the clinical application of phage–antibiotic combination therapy.

**FIGURE 1:**
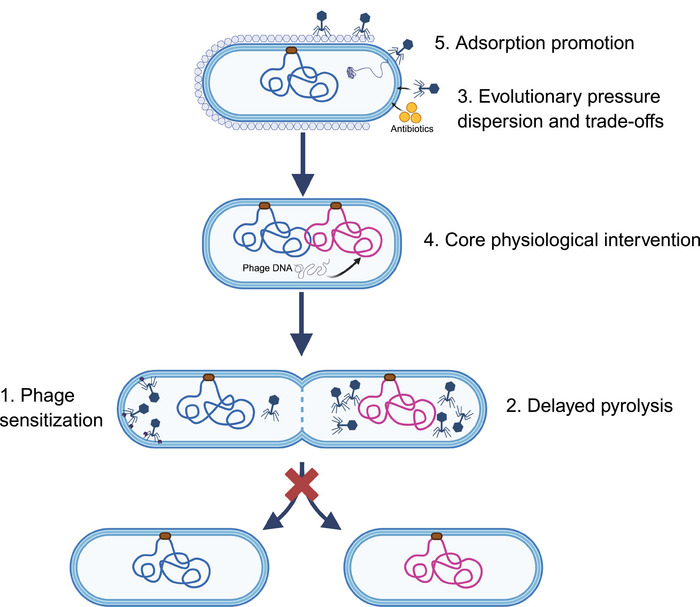
Mechanism of phage–antibiotic synergy. (1) Antibiotic‐induced disruption of the peptidoglycan layer sensitizes bacteria to phage‐encoded lytic enzymes. (2) Antibiotics can induce bacterial filamentation, delaying bacterial lysis and increasing phage burst size. (3) The combined pressure from phage and antibiotic treatment reduces the emergence of bacterial tolerance. (4) Phage infection disrupts core metabolic processes and transport systems, weakening intrinsic antibiotic resistance. (5) Phage‐mediated depolymerization of extracellular polymeric substances (EPS) enhances antibiotic diffusion and penetration.

Multiple studies indicate that the synergistic effect of phage–antibiotic combinations is not merely additive, but results from multitarget interventions that enhance therapeutic efficacy. The mechanisms underlying phage–antibiotic synergy include several key aspects (Figure 1). First, certain antibiotics, such as β‐lactams and quinolones, inhibit bacterial cell division, leading to filamentation and disruption of the peptidoglycan layer. This alteration makes bacterial cells more susceptible to phage‐encoded lytic enzymes [88]. Second, some antibiotics can induce delayed bacterial lysis, providing an extended window for intracellular phage replication, which results in a higher burst size and larger plaque formation. An in vitro study demonstrated that combining phages targeting *S. aureus* with antibiotics, including clarithromycin, linezolid, cefotaxime, tetracycline, and ciprofloxacin, significantly enhanced phage progeny production [89]. Third, antibiotics can reduce the emergence of phage‐resistant bacteria. From an evolutionary perspective, applying dual selective pressures (phage and antibiotic) reduces the likelihood of resistance development compared with using a single pressure [90]. This rationale aligns with the use of phage cocktails over mono‐phage formulations. Additionally, from a fitness cost perspective, even if bacteria develop resistance to phages, they may still be vulnerable to antibiotics due to regained susceptibility [91]. For instance, in an in vitro study by Wang et al. [92] on colistin and *Acinetobacter baumannii* (*A. baumannii*) phage Phab24, phage‐resistant bacteria that evolved in the absence of antibiotics showed increased sensitivity to colistin. Qin et al. [93] employed multiomics and Raman spectroscopy to demonstrate, at both population and single‐cell levels, that a phage–ceftazidime combination suppressed bacterial metabolic activity and increased the fitness cost of phage‐resistant variants, altering the evolutionary trajectory of bacterial populations. Fourth, phage infection can directly interfere with core physiological processes in bacteria, such as energy metabolism and material transport, thereby compromising the intrinsic resistance mechanisms of the bacterial host. Kraus et al. [82] demonstrated that phage ΦBP‐AMP1 downregulated the expression of genes encoding multidrug efflux pumps, as well as genes associated with the energy supply for these pumps, leading to intracellular accumulation of antibiotics. Importantly, the efflux pumps were not the receptors for phage ΦBP‐AMP1, indicating a synergistic mechanism distinct from adaptive fitness trade‐offs. Finally, the limited efficacy of certain antibiotics against specific bacterial strains is partly due to the barrier formed by bacterial extracellular polymeric substances (EPS). Phage‐encoded depolymerases can degrade structural or capsular polysaccharides, enhancing antibiotic diffusion and penetration. For example, *P. aeruginosa* phages may encode alginate lyases [94], which degrade bacterial biofilms and facilitate the diffusion of aminoglycoside antibiotics to the bacterial surface [95], thereby promoting both bactericidal activity and biofilm eradication.

Despite the focus on phage–antibiotic synergy, antagonistic interactions between the two are often overlooked. In some cases, combination therapy may lead to suboptimal outcomes compared with either treatment alone [96, 97]. This may be due to the action of certain antibiotics. As most phage progeny production relies on the host bacterial RNA polymerase, rifampicin, which binds to bacterial RNA polymerase, inhibits phage replication, resulting in antagonistic effects [97]. These findings highlight the importance of thoroughly evaluating interactions between phages and various antibiotics prior to clinical application.

### 2.4 Biomaterial‐Enhanced Phage Therapy

Building on the synergistic potential of phage–antibiotic combinations, advanced material‐based strategies present transformative opportunities to improve the precision, stability, and efficacy of phage therapy (Table 1). These platforms not only protect phages in challenging environments, such as the gastrointestinal tract or wound sites, but also enable deep‐tissue targeting and intracellular delivery, thereby expanding the therapeutic applications of phages to both infectious and noninfectious diseases.

**TABLE 1:** Route‑dependent biomaterial‑phage delivery strategies.

Applications	Bacteria	Delivery method	Biomaterial	Animal studies	Encapsulation efficiency	Dimensions	References
Intestine	*Salmonella*	p.o.	Eudragit L100‐55	Yes	93.7%	—	[98]
	*Streptococcus Gallolyticus*	i.g.	Eudragit L100‐55	Yes	—	290.5 nm, 289.0 nm	[99]
	*E. coli*	p.o.	Alginate‐based hydrogels	Yes	—	—	[100]
	*Salmonella*, *E. coli*,*Shigella.flexneri*	p.o.	Chitosan‐based hydrogels	Yes	80 ± 5.5%	298.2 ± 2.45 nm	[101]
	*Fusobacterium nucleatum*	i.p.	AgNPs	Yes	—	800 nm	[102]
Wound	MRSA	TOP	Liposome	Yes	87%	212 nm	[103]
	Ab	TOP	Chitosan‐based hydrogels	Yes	91.30 ± 1.94%	1.291 ± 0.535 µm	[104]
	Kp	TOP	Chitosan‐based hydrogels	Yes	82.44 ± 1.31%	1.96 ± 0.51 µm	[105]
	MRSA, Pa	TOP	Chitosan‐based hydrogels	Yes	87.56 ± 1.03, 79.52 ± 0.84	2.846 ± 0.288 µm	[106]
	Pa	Ex vivo	Gelatin‐based hydrogel	No	—	—	[107]
	MRSA	TOP	Poloxamer 407 hydrogel	Yes	—	275 nm	[108]
	Pa	TOP	Quantum dot (QD)	Yes	—	—	[109]
	Ab	TOP	Nile blue dyes (NB)	Yes	—	—	[110]
	*E. coli*, Pa	SC	Pd nano‑enzymes	Yes	—	—	[111]
	Pa	TOP	RuO_2_ nanozyme	Yes	70.72%	—	[112]
Bone	Pa	TOP	PEG‐4MAL‐based hydrogels	Yes	—	—	[113]
Lung	Kp	i.p.	Liposome	Yes	92%	120.7 ± 2.7 nm	[114]
	Pa	inh.	PLGA	Yes	—	8.0 µm	[115]
	*S. aureus*	inh.	PLGA	Yes	—	6.25 ± 0.38 µm	[116]
	MRSA	inh.	PLGA	Yes	97.3 ± 0.7%	4.62 µm	[117]
	Pa	In vitro	Liposome	No	58.33 ± 6.02%	171.13 ± 2.04 nm	[118]
	Pa	In vitro	Liposome	No	77.9 ± 1.48%, 83.9 ± 4.23%	301 ± 35.8 nm, 651 ± 14.3 nm	[119]
	*E. coli*	ITI	Pd nano‑enzymes	Yes	—	—	[111]
Cell entry	*Salmonella*	In vitro	Hydroxyapatite (HA)	No	—	400 nm	[120]
	*Salmonella*	i.g.	Cationic polymers PEI	Yes	—	289.4 nm	[121]

Abbreviations: Ab, *Acinetobacter baumannii*; i.g., oral gavage; inh., inhalation administration; i.p., intraperitoneal injection; ITI, intratracheal instilled; Kp, *Klebsiella pneumoniae*; MRSA, methicillin‐resistant *Staphylococcus aureus*; Pa, *Pseudomonas aeruginosa*; p.o., oral administration; SC, subcutaneous Injection; TOP, topical administration.

#### 2.4.1 The Challenge of Oral Phage Delivery and Encapsulation Strategies

As a biological agent, phage stability and activity are critical concerns. Extensive characterization studies show that phages can tolerate a pH range of approximately 3–10. However, the gastric environment typically has a pH lower than 2, which is unfavorable for oral phage delivery. Additionally, trypsin and bile present in the intestine can damage the phage capsid [122, 123]. Enteric‐coated tablets, a proven formulation for intestinal drug delivery, have been applied to phage delivery. Eudragit L100‐55 (also known as ACRYL‐EZE) effectively protects phages and enables their precise release in the intestine. This polymer has been used to treat *Salmonella*‐induced bacterial enteritis [98] and can also encapsulate phage cocktails (DNPs@P) to target and eliminate *Streptococcus gallolyticus* (Sg) from the gut, preventing Sg‐associated colorectal cancer [99]. Hydrogels are another widely used platform for oral phage delivery. Hsu et al. utilized an alginate‐based hydrogel coating to encapsulate *Salmonella* phages, which remain intact under acidic stomach conditions but degrade at neutral pH in the intestine, releasing highly active phages and significantly improving oral bioavailability and therapeutic efficacy [100]. In another study, chitosan‐based hydrogels encapsulated multiple phages for oral delivery to treat diarrhea caused by *Escherichia coli* (*E. coli*), *Shigella*, and *Salmonella*. In comparison with antibiotic therapy, the encapsulated phages effectively reduced pathogen detection in fecal samples [101].

#### 2.4.2 Protective Biomaterials for Phage Therapy in Complex Wounds

The infection microenvironment often features neutrophil infiltration, decreased pH, and elevated H_2_O_2_ levels [124, 125], all of which can impair phage lytic activity. In these conditions, biomaterials can protect or enhance phage efficacy. Research has shown that hydrogels composed of various matrices, including liposomes [103], PEG‐4MAL [113], chitosan [104, 105, 106], and gelatin [107], can shield phages in complex infected wound environments, effectively targeting MDR pathogens such as methicillin‐resistant *S. aureus* (MRSA), *P. aeruginosa*, *A. baumannii*, and *K. pneumoniae*. Characterization of encapsulated phage formulations indicates that encapsulation efficiency generally exceeds 80%, with these formulations demonstrating strong bactericidal effects in animal models. Phage–polymer nanoassemblies encapsulated in Poloxamer 407 hydrogel retained the infectivity of native phages while showing enhanced biofilm penetration compared with free phages [108].

Ran [110] and Wang [109], along with their colleagues, have pioneered the covalent conjugation of sulfur‐modified cationic photosensitizers (NB) and quantum dots (QD) onto phage surfaces. This innovative approach retains the natural targeting and lytic properties of phages while enabling the generation of substantial reactive oxygen species (ROS) upon near‐infrared light excitation. The ROS produced lead to oxidative damage and membrane disruption in bacteria. This strategy has been successfully applied to biofilm eradication and significantly accelerated wound healing in murine infection models. During the synthesis of these modified phages, careful control over the amount of conjugated biomaterial is essential to avoid excessive coupling to the phage tails, which could hinder host recognition [126]. These composite antimicrobial systems not only enhance the lytic efficiency of phages but also extend their ability to target and eliminate complex bacterial communities, such as biofilms, highlighting the synergistic potential of chemical modifications in advancing phage therapeutic efficacy.

Nanoenzymes have also been utilized to chemically modify phages, harnessing their intrinsic peroxidase activity to catalyze the transformation of elevated hydrogen peroxide and hydrogen ions found in infected microenvironments into active oxygen species, thus exhibiting precise targeting and potent antimicrobial effects [127, 128]. For instance, palladium (Pd) nanoenzymes are conjugated to M13 phages via N‐hydroxysuccinimide groups, forming the Phage@Pd hybrid system [111]. This system exploits the peroxidase‐like activity of Pd to catalyze the conversion of hydrogen peroxide and protons into hydroxyl radicals (·OH), achieving site‐specific elimination of *E. coli*. Interestingly, Phage@Pd also showed activity against the non‐host bacterium *S. aureus*, extending its bactericidal effect. This broad‐spectrum activity is attributed to the filamentous structure of the M13 phage, which facilitates entanglement with bacterial surfaces, as confirmed by transmission electron microscopy. In animal models of acute pneumonia and subcutaneous biofilm infections, Phage@Pd demonstrated superior antibacterial efficacy compared with phages alone, alleviating tissue inflammation [111]. The system showed good safety in vivo, with no significant inflammation or organ damage, even at high doses. However, its peroxidase activity is most effective at a pH of 6.0 and ceases under neutral or alkaline conditions, limiting its applicability in the gut [129]. Additionally, body proteins can block the active sites of Pd, reducing its activity—this is particularly concerning for patients with weakened immune systems.

Expanding on this integrated strategy, Wang et al. developed a system based on RuO_2_ nanozyme, which also functions as a ROS scavenger. When combined with *P. aeruginosa* phage PA3, this system achieves dual‐mode antibacterial synergy. While the phage directly lyses bacterial cells, RuO_2_ mitigates infection‐related inflammation and oxidative damage, supporting host immune clearance mechanisms and reducing the risk of resistance development [112]. This combined approach not only enhances bactericidal efficacy but also promotes tissue repair, offering a targeted and immunomodulatory strategy for treating persistent bacterial infections. The fusion of phage specificity with nanozyme catalysis presents a promising direction for the future of antibacterial therapy.

#### 2.4.3 Biomaterial‑Enabled Phage Delivery to Deep Tissue Infections

Liposomes are a widely used drug delivery system, particularly for pulmonary administration. Their drug release mechanism within the lungs depends on structural responsiveness to the local microenvironment, enabling controlled release at the infection site. pH‐sensitive liposomes undergo structural changes in the acidic pulmonary environment, accelerating drug release [130]. Thermosensitive formulations, such as ThermoDox, release their contents under localized hyperthermia [131], while enzyme‐responsive systems are activated by overexpressed pulmonary enzymes [132]. These strategies facilitate targeted and controlled drug release, enhancing treatment efficacy.

A decade ago, Singla et al. compared intranasal and intraperitoneal delivery of liposomal phages against *K. pneumoniae* in mice [114]. Theoretically, intranasal delivery allows direct targeting of pulmonary infection sites, while intraperitoneal delivery enables liposomes, smaller than 200 nm, to extravasate into the lung interstitium through increased vascular permeability induced by infection or inflammation [133]. Surprisingly, intraperitoneal injection resulted in significantly better therapeutic outcomes. Subsequent studies on inhaled phages have been limited, likely due to phage degradation during nebulization, low encapsulation efficiency, and suboptimal particle size [134]. Recent advances show that liposome encapsulation can effectively protect phages during nebulization and enable controlled release in simulated lung fluid. Additionally, liposome coating significantly reduces phage uptake by human lung epithelial cells [118, 119]. These improvements uncover the potential of liposomal technology in inhaled phage therapy and highlight the need for large‐scale studies to evaluate its efficacy against pulmonary infections.

Agarwal et al. proposed an alternative delivery strategy using porous poly(lactic‐co‐glycolic acid) (PLGA) microparticles, composed of polylactic acid and polyglycolic acid. Instead of encapsulating phages within PLGA microparticles, they were deposited onto the porous surface. Phage‐loaded PLGA microparticles were delivered via dry powder inhalation to the lung infection site, significantly reducing *P. aeruginosa* burden and improving survival in a mouse model [115]. Further research demonstrated that phage‐loaded PLGA microparticles effectively alleviated acute lung infections caused by *S. aureus* and facilitated the delivery of phage cocktails targeting multiple bacterial species [116]. Building on this approach, Liu's team modified the porous PLGA microparticles with indocyanine green and loaded them with engineered phages for inhalable dry powder delivery. This system achieved precise targeting and synergistic antibacterial effects against MRSA in deep lung tissues [117].

Controlling particle size is critical for enhancing delivery efficiency. For inhalation administration, particles with an aerodynamic diameter of less than 5 µm can ensure sufficient deposition in the alveoli, avoiding rapid clearance by alveolar macrophages [115, 135]. This size range helps prolong phage residence time at the infection site, thereby enhancing therapeutic efficacy. When using PLGA for phage encapsulation, it is essential to avoid organic solvents to prevent phage protein denaturation [116]. Other polymeric materials, such as polylysine, polyarginine, and poly(allylamine), may also inhibit phage activity [136]. Therefore, thorough validation of material selection is essential. Systematic evaluation of phage compatibility with encapsulation solvents and the retention of phage titer postencapsulation is necessary for successful practical application [137].

#### 2.4.4 Strategies for Phage‐Based Clearance of Intracellular Bacteria

Phages possess a negative charge on their capsids [138], similar to the surface charge of eukaryotic cells [139], which hinders their ability to enter eukaryotic cells and clear intracellular pathogens. To overcome this barrier, Meng et al. used the cationic polymer polyethylenimine (PEI) to selectively coat the phage head. This modification reverses the phage's surface charge to positive, promoting its entry into eukaryotic cells through endocytosis [121]. According to the proton sponge hypothesis, the PEI@P complex enters early endosomes, bypassing lysosomal degradation. To maintain ion balance, chloride influx triggers endosomal osmotic swelling and membrane rupture [140], enabling the PEI@P complex to escape into the cytoplasm and target intracellular *Salmonella*. Animal studies demonstrated that oral administration of PEI@P significantly reduced the migration of intestinal *Salmonella* to the liver and spleen [121]. In another approach, A. Fulgione et al. utilized hydroxyapatite (HA) nanocrystals as carriers for phage SRφ1. The positive surface charge of HA neutralizes the negative charge on the phage, minimizing electrostatic repulsion [120]. This system provides both physical protection and charge modulation, facilitating the phage's ability to cross cellular barriers and function intracellularly.

Although cationic polymers like PEI showed no significant cytotoxicity in preliminary studies using MODE‐K intestinal epithelial cells and murine models, further safety evaluations are essential to fully establish their safety profile [121]. Emerging evidence suggests that internalized phage DNA may trigger immune responses, including the production of anti‐inflammatory factors such as Type I interferons and interleukin‐4 [141]. This potential immunogenicity highlights the need for a comprehensive assessment of cytotoxicity profiles to ensure the safety of therapeutic applications.

#### 2.4.5 Biomaterial‑Assisted Phage Therapy for Noninfectious Diseases

In addition to their antibacterial applications, phages are being developed for the treatment of noninfectious diseases caused by commensal bacteria. Specific *K. pneumoniae* clades have been linked to the exacerbation of inflammatory bowel disease (IBD) [142]. A tailored five‐phage cocktail effectively suppressed these strains in murine models, alleviating intestinal inflammation. Similarly, in nonalcoholic fatty liver disease associated with high alcohol‐producing *K. pneumoniae*, targeted phage therapy reduced steatohepatitis, improved metabolic profiles, and restored gut–liver axis homeostasis without significant dysbiosis [143].

However, elevated ROS levels in IBD patients can impair phage therapy efficacy [144]. A novel approach integrating DNA nanoscaffolds (DNPs) with *Sg*‐targeting phages (P‐Sg) has been proposed. In this design, DNPs scavenge ROS, while the conjugated phages deliver the nanoscaffolds to inflamed sites, leading to targeted bacterial lysis. To enhance gastrointestinal stability, DNPs@P were encapsulated in Eudragit L100‐55, enabling colon‐specific release. This formulation effectively prevented colitis‐associated tumorigenesis, demonstrating proof‐of‐concept for a new class of targeted IBD therapies [99].

Silver nanoparticles (AgNPs) exert antibacterial effects by releasing silver ions that disrupt bacterial membranes, inhibit enzymatic activity, and induce ROS generation [145, 146]. Recent studies have integrated AgNPs with phages to enhance specificity and efficacy. AgNPs were electrostatically assembled onto M13 phages targeting *Fusobacterium nucleatum*, enabling targeted bacterial clearance and immune activation in colorectal cancer models [102]. However, AgNPs raise safety concerns due to their potential to accumulate in organs, induce oxidative stress, and cause cytotoxicity [147]. To mitigate these risks, recent strategies have focused on improving biocompatibility and controllability. For example, encapsulating AgNPs in biodegradable, thermo‐sensitive hydrogels allows for controlled silver release, reducing local accumulation while preserving antibacterial activity [148]. Additionally, synthesizing AgNPs with diameters greater than 20 nm has been shown to reduce cytotoxicity while maintaining their antimicrobial function [149]. This size‐dependent optimization balances antimicrobial penetration with biosafety considerations.

#### 2.4.6 Synergistic Integration of Phage‐Derived Enzymes and Biomaterials

Phage‐encoded endolysins, which degrade bacterial peptidoglycan, exhibit potent and specific bactericidal activity. However, their clinical application is hindered by poor stability, a short half‐life, and immunogenicity [150, 151]. Encapsulation of endolysin Cpl‐1 in chitosan nanoparticles enhances bioavailability and enables sustained release, significantly reducing *Streptococcus pneumoniae* (*S. pneumoniae*) loads in a murine pneumonia model [152]. Similar approaches have been applied to deliver endolysins targeting Gram‐positive bacteria, such as LysMR‐5[153] and LysSYL [154], both expressed by *S. aureus*. However, the outer membrane of Gram‐negative bacteria poses a barrier to endolysin access. Cationic liposomes can encapsulate endolysins, promoting interaction with the bacterial envelope while shielding them from degradation, thus demonstrating broad activity against pathogens such as *Salmonella*, *E. coli*, and *P. aeruginosa* [155, 156, 157]. Environmentally responsive systems further enhance targeting. pH‐sensitive hydrogels release staphylococcal endolysins LysSYL specifically in acidic wound environments [154], while thermosensitive hydrogels deliver endolysin LysP53 against *A. baumannii* in wound models [158]. These smart materials facilitate healing and reduce bacterial burden.

Phage‐encoded depolymerases also play a critical role in synergizing with biomaterials. By degrading surface polysaccharides, they expose underlying bacterial targets, enhancing antimicrobial penetration [159]. When integrated into delivery platforms, depolymerases can pretreat biofilms or resistant bacteria, clearing the way for endolysins or antibiotics [160]. Immobilizing depolymerases on nanoparticles enables specific recognition and degradation of bacterial capsules [161].

Phage‐derived enzymes hold substantial promise for clinical applications (Table 2). Encapsulation strategies can preserve enzymatic activity, enhance tissue‐specific targeting, and prolong in vivo retention. However, additional in vivo studies are needed to optimize delivery platforms, confirm therapeutic safety, and assess scalability.

**TABLE 2:** Biomaterial‑enabled delivery of phage‑derived enzymes.

Enzyme	Bacteria	Delivery method	Applications	Animal studies	Encapsulation efficiency	Dimensions	References
Chitosan nanoparticles‐Cpl‐1	*S. pneumoniae*	i.n.	Lung	Yes	84.51 ± 4.21 %	100 nm	[152]
Chitosan nanoparticles‐ LysMR‐5	MRSA	—	In vitro	No	62 ± 3.1 %	<100 nm	[153]
Self‐assembling peptide hydrogels‐LysSYL	MRSA	TOP	Wound	Yes	85.7 %	13.2 ± 0.5 nm	[154]
Liposome‐ BSP16Lys	*Salmonella*, *E. coli*	—	In vitro	No	35.27 %	303 nm	[155]
Liposome‐Pa7	Pa	—	In vitro	No	33.33 %	151 ± 6 nm	[157]
Liposome‐Pa119	Pa	—	Lung	No	32.30 %	149 ± 4 nm	[157]
Liposome‐ Lysqdvp001	*V. parahaemolyticus*	—	In vitro	No	56.65–62.38 %	1088.73–1579.00 d nm	[156]
Thermosensitive hydrogel‐ LysP53	Ab, *S. aureus*	TOP	Wound (ex vivo)	No	—	—	[158]
Gold nanoparticles‐ depolymerases	Ab	—	In vitro	No	—	32 ± 4 nm	[160]

Abbreviations: *V. parahaemolyticus*, *Vibrio parahaemolyticus*; i.n., intranasal delivery; TOP, topical administration.

When selecting biomaterials for phage delivery, it is crucial to consider the physiological structure and microenvironment of the target organs and tissues, along with the specific requirements of the chosen administration route. This ensures the optimization of both delivery efficiency and biocompatibility. While promising therapeutic potential has been demonstrated in vitro and in animal models, clinical translation faces significant hurdles, including formulation stability, scalable production, long‐term safety, and regulatory approval. As a result, the practical clinical application of these strategies remains a considerable distance away.

### 2.5 Design and Application of Engineering Phages

Currently, nearly all phages used in clinical therapy are derived from the environment, representing wild‐type phages with high specificity but limited host range. To address this limitation, researchers are employing complementary strategies such as constructing phage cocktails and combining phages with other antimicrobial agents. Additionally, genetic engineering is being applied to modify the phages themselves, aiming to develop engineered variants with broader host ranges and enhanced bactericidal efficacy in challenging environments, such as harsh conditions or biofilms. For instance, Feng et al. replaced the RBP of phage RCIP0035, originally targeting KL2 *K. pneumoniae*, with one from another phage targeting KL57. This modification caused the phage to lose its lytic ability against KL2 *K. pneumoniae* but gain infectivity against KL57. Moreover, by replacing the RBP with one that exhibits multiple capsular lytic activities, the host range of the recombinant phage was broadened [162]. Similarly, Song et al. achieved precise modulation of phage host range by swapping the tail fiber genes of phage PHB20 [163]. In another approach, Huss et al. used metagenomics to identify key mutation combinations. Introducing multiple mutations into the phage genome increased the positive charge of the RBP, allowing it to bind specifically to the LPS of *E. coli* O121, a strain resistant to lysis by both wild‐type and single‐amino‐acid‐mutant T7 phages [164]. Furthermore, a phage cocktail named SNIPR001, composed of four engineered phages, demonstrated over 90% coverage against 382 *E. coli* strains from various countries. In mouse models, it significantly reduced the intestinal burden of *E. coli*, particularly resistant strains [165].

Temperate (lysogenic) phages are generally excluded from clinical use due to their potential to integrate into the bacterial genome, which carries the risk of transferring antibiotic resistance or virulence genes [166]. However, both in vitro and in vivo studies have demonstrated that temperate phages from various bacterial species can be converted into obligately lytic phages by removing their lysogeny modules through genetic engineering [167, 168, 169, 170], thus enabling their safe and effective use as antimicrobial agents. Recent advances in phage genetic editing techniques have moved from traditional homologous recombination to more sophisticated methods, including the CRISPR/Cas system [171, 172, 173] and BRED (bacteriophage recombineering of electroporated DNA) [174]. Utilizing CRISPR/Cas technology, Bikard et al. [175] developed a novel bactericidal strategy involving an engineered *S. aureus* temperate phage, φMN1. This phage delivers a CRISPR–Cas module targeting specific *S. aureus* virulence genes, selectively killing strains harboring these genes without triggering excessive endotoxin release. A similar strategy has shown promising results in treating *Clostridioides difficile* infections [176]. By deleting key lysogeny genes and engineering the temperate *C. difficile* phage with a CRISPR module targeting *C. difficile* genomic sequences, the resulting lytic phage reduced *C. difficile* burden in vivo and alleviated disease severity.

The expansion of host range for lytic phages and the reengineering of lysogenic phages both focus on concentrating desired traits within existing phages. However, for bacterial pathogens for which no suitable phages are available, the synthetic construction of phages becomes essential. Currently, in vitro phage synthesis remains in its early stages [177]. This process begins with the in vitro synthesis of the phage genome, which requires deciphering the functional connections between phage and bacterial genomes from extensive datasets of phage–bacteria interactions and their genomic annotations. While advances in artificial intelligence [178, 179] have supported this task, the approach remains challenging due to difficulties in reliably synthesizing large DNA molecules using traditional organic chemistry methods [180, 181]. Even if the phage genome is successfully synthesized, it must be reconstituted into an active state. This is typically achieved by transforming the genome into *E. coli* [182] or using a cell‐free transcription–translation system [165, 183, 184] to reactivate the complete genome, a process known as phage genome reboot. One of the main obstacles to advancing de novo phage synthesis technology is improving the efficiency of this reboot process. Nonetheless, with anticipated reductions in gene synthesis costs, the development of high‐fidelity polymerases, and the establishment of universal in vitro reboot systems, these challenges are expected to be overcome in the near future.

The pace of phage genome editing has advanced alongside the development of phage therapy, with several engineered phages already progressing to clinical trials. In 2021, Locus Biosciences announced the completion of a Phase 1b clinical trial and the initiation of a Phase 2 trial for LBP‐EC01, a CRISPR–Cas3‐enhanced phage (crPhage) targeting *E. coli*, a common cause of UTI [22]. Trial results demonstrated that LBP‐EC01 effectively reduced the levels of susceptible bacteria in the bladders of patients infected with *E. coli*, with the CRISPR–Cas3 technology enhancing the phage's bactericidal activity. The trial also confirmed the safety and tolerability of LBP‐EC01, marking the first completed randomized, placebo‐controlled trial of a recombinant phage therapy.

However, engineered phages carry inherent risks. Modifications to phage genome sequences may impact their infectivity and replication capabilities [185], and their interactions with the human immune system are not yet fully understood [186, 187]. Additionally, the potential environmental release of gene‐edited phages raises concerns about their effects on native bacterial populations and microbial communities [188]. Despite several engineered phage candidates being authorized through the US FDA's Emergency Investigational New Drug program [189], the current regulatory framework lacks comprehensive safety and efficacy data for both temperate and engineered phages. Ethical considerations remain unresolved, leading regulatory bodies to favor strictly lytic phages for clinical applications. Nonetheless, intelligent phage‐based formulations offer significant promise in overcoming the limitations of conventional antibiotics, ushering targeted therapy into a new, designable, and controllable phase.

## 3 Phage Applications in Nosocomial Infection Prevention and Control

**FIGURE 2:**
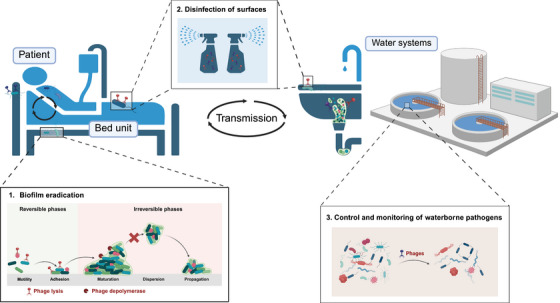
Use of phages in the prevention and control of nosocomial infections. (1) Biofilm eradication through targeting biofilm maturation and propagation by phage‐mediated lysis and depolymerase activity. (2) Disinfection of hospital wards to eliminate pathogenic reservoirs from high‐touch surfaces. (3) Surveillance and control of waterborne pathogens within hospital water systems.

In the context of hospital infection control, environmental colonization and pathogen transmission present a persistent and complex challenge. Specifically, bacterial biofilms, hospital environmental surfaces, and water distribution systems act as critical reservoirs and dissemination points for MDR organisms (MDROs) and ARGs. This section examines the research progress and potential applications of phages in these three key areas (Figure 2), while also addressing the challenges they present, such as the risk of HGT and the evolution of host resistance.

### 3.1 Biofilm Eradication

Bacterial biofilms are the underlying cause of many chronic infections, accounting for approximately 65% of such cases [190]. They typically form in response to antibiotic exposure or competition with other bacterial strains and are commonly found on the surfaces of medical devices and equipment, acting as persistent sources of contamination [191]. Biofilms consist primarily of microorganisms embedded within an EPS matrix, which is mainly composed of polysaccharides, along with proteins, lipids, small amounts of nucleic acids, and inorganic materials. In biofilms, bacteria are tightly encased by the self‐secreted EPS matrix, which impedes the penetration of antibiotics and disinfectants, leading to recurrent and persistent infections. Additionally, restricted diffusion of oxygen and nutrients slows bacterial metabolism and growth, causing a large proportion of cells to enter a dormant or nondividing state. Antibiotics are less effective against these dormant bacteria [192], a phenomenon first described in 1944 by Joseph Bigger as a “dormant, nondividing phase” [193]. Moreover, biofilm communities are often polymicrobial, consisting of a micro‐ecosystem formed by various bacteria, fungi, and other microorganisms. These microbes can interact synergistically, enhancing the biofilm's environmental adaptability and resistance to stress, which complicates clinical eradication [194].

Research indicates that phages and their encoded enzymes can serve as promising alternatives for controlling and preventing biofilms. Phage‐mediated biofilm lysis begins with the degradation of the EPS matrix by depolymerases. These enzymes, encoded by phages, specifically target and break down the polysaccharide components of bacterial cell walls and biofilm matrices [195]. Hernandez et al. [196] identified phage Petty, capable of infecting both *A. baumannii* and *A. nosocomialis*. The putative tail fiber protein, Dpo1, exhibited depolymerase activity. The researchers cloned the gene encoding Dpo1, overexpressed, and purified the recombinant protein, demonstrating that it degraded EPS and significantly reduced biofilm viscosity. Similarly, a study by Shahed‐Al‐Mahmud et al. [197] showed that another *A. baumannii* depolymerase, TSP, not only degraded biofilms but also inhibited bacterial colonization on medical catheter surfaces, thereby preventing bacterial adhesion to abiotic surfaces.

Following the initial breakdown of the EPS matrix by depolymerases, phages can penetrate the dense biofilm and reach the protected bacterial cell surfaces. The high bacterial density within biofilms leads to a correspondingly high rate of phage replication, with the large number of progeny phages further contributing to the localized lysis of the biofilm [198]. Recent studies have shown that phage Paride can directly target and kill antibiotic‐tolerant dormant cells of *E. coli* or *P. aeruginosa* through lytic replication. Although the burst size is reduced and the latent period extended to 2.5 h compared with actively growing cells, the activity remains significant [199]. Furthermore, when combined with meropenem, phage Paride achieved complete eradication of deeply dormant *P. aeruginosa*, whereas meropenem alone, or in combination with other antibiotics such as ciprofloxacin, did not yield the same result. Mechanistic studies revealed that this synergistic effect arises from a phage‐induced “lysis of bystanders” phenomenon, wherein phage‐induced lysis of bacterial cells exposes drug‐sensitive bacteria to the antimicrobial agent, leading to their effective clearance. It has been established that the bacterial stringent response alarmone (p)ppGpp and the master stress regulator RpoS play pivotal roles in regulating the dormant state and antibiotic tolerance. Notably, Paride's ability to replicate in nongrowing hosts is directly linked to the bacterial dormancy state, highlighting its unique mechanism for targeting persister cells [199].

Cystic fibrosis, a genetic disorder, is frequently complicated by recurrent lung infections, which are a major cause of morbidity and mortality in patients. *P. aeruginosa* is the predominant pathogen, often existing in a biofilm state [200]. Phage therapy has emerged as a promising novel therapeutic approach for treating cystic fibrosis patients with drug‐resistant *P. aeruginosa* infections. A study involving cystic fibrosis patients demonstrated a significant reduction in sputum *P. aeruginosa* load following phage treatment [56]. Within 5–18 days of therapy, bacterial counts decreased by up to 10^4^‐fold, far surpassing the typical 10^2^–10^3^‐fold reduction achieved with conventional antibiotics. Long‐term follow‐up also indicated a continued reduction in bacterial burden. This highlights the ability of phages to effectively penetrate biofilm structures and eliminate persister cells encased within the EPS matrix [191], offering a promising therapeutic strategy for MDR infections.

### 3.2 Disinfection of Hospital Environmental Surfaces

Numerous studies have highlighted the bidirectional flow of MDROs between patients and their surrounding environments, particularly in intensive care units (ICUs). Environmental sampling around the beds of MDRO‐infected patients consistently tests positive, and patient transfers between units further accelerate the spread of resistant bacteria [201, 202], creating complex transmission networks and emphasizing the critical role of the environment as an intermediary. The persistence and spread of antibiotic resistance in these settings are primarily driven by HGT, facilitated by mobile genetic elements (MGEs) such as plasmids and transposons [203]. By combining metagenomic sequencing with nanopore long‐read sequencing for systematic hospital surveillance, Chng and colleagues [204, 205, 206] demonstrated that MDROs gain a clear selective advantage in the hospital environment, persisting for extended periods, spreading widely, and showing high genetic similarity to global clinical isolates. Notably, plasmids carrying ARGs were detected in over 85% of sampling sites [204], highlighting the widespread distribution of these mobile elements throughout hospital environments.

Conventional chemical disinfection methods exhibit significant limitations in controlling MDR pathogens on hospital surfaces [207]. An interventional study conducted in an ICU found that increasing the frequency of environmental cleaning to twice daily did not significantly reduce the acquisition rate of carbapenem‐resistant *A. baumannii* (CRAB) [208]. Additionally, Duran et al. observed that persistent *S. aureus* clusters from hospital environments were not only enriched with virulence genes but also harbored three distinct disinfectant‐resistance genes [209]. This suggests that chemical disinfectants, through selective pressure, may inadvertently promote the evolution of resistant strains, creating a harmful cycle. While ultraviolet (UV) sterilization effectively inactivates pathogens on exposed surfaces, it requires unoccupied spaces for operation, and its limited penetration capacity hinders its ability to eliminate pathogens hidden in crevices or biofilms [210, 211], restricting its frequency and scope of practical application in clinical settings.

Phages and combined strategies have shown significant effectiveness in hospital environmental decontamination. Chen et al. demonstrated that the intervention of phage aerosols in an ICU significantly reduced the incidence of newly acquired CRAB, from 8.57 to 5.11 cases per 1000 patient‐days. During a 3‐year follow‐up, the infection rate in the ICU remained consistently lower at 4.4 cases per 1000 patient‐days, a significant reduction compared with the 8.9 cases per 1000 patient‐days in the control ward without intervention [212]. These findings were further supported by another study, where intervention with a rationally designed phage cocktail led to a significant decrease in CRAB percentage in the ICU from 65.3 to 55%. Additionally, during the phage intervention period, antimicrobial usage, excluding imipenem, was significantly reduced, highlighting the added benefit of decreasing antimicrobial consumption [213]. Yinghan et al. further corroborated these findings. A mixture of three phages targeting ST11‐KL47 and ST11‐KL64 Carbapenem‐resistant *K. pneumoniae* (CRKP) effectively reduced pathogen levels within 24 h, with sustained effects. While bacterial loads on surfaces showed no significant change 6 h after chemical disinfectant treatment, phage treatment led to reductions from 1.31 × 10^4^ copies/µL and 1.48 × 10^4^ copies/µL to 3.49 × 10^3^ copies/µL and 4.02 × 10^3^ copies/µL in two bed units after 24 h, maintaining lower levels for up to 48 h. 16S rRNA sequencing analysis revealed no significant alteration in the structure of nontarget microbial communities, indicating that phage intervention effectively reduced the relative abundance of target pathogens while preserving the overall diversity and stability of the environmental microbiota [214]. Regarding synergistic effects of combined strategies, D'Accolti et al. explored the use of phages in combination with probiotics. The study found that daily nebulized phage application resulted in an additional 97% reduction in *Staphylococcus* load on treated surfaces compared with probiotic‐based disinfection alone [215]. This highlights a strong synergistic effect between the two approaches, which retains the stability benefits of probiotic cleaning while enabling rapid and targeted clearance of specific pathogens through phage activity.

The impact of chemical disinfectants on phages varies significantly. Benzalkonium chloride at 0.5% wt/vol exhibits a pronounced inhibitory effect on phages [216], while triclosan and chlorhexidine have minimal impact on their viability [217]. This variation is not only dependent on the chemical properties of the disinfectants but also shows a concentration‐dependent characteristic. Earlier studies using lower concentrations of benzalkonium chloride did not show significant inhibition [217, 218], whereas higher concentrations commonly used in clinical practice clearly impair phage activity. Notably, most phages exhibit strong tolerance to traditional disinfectants such as 70% alcohol and hydrogen peroxide. Studies in food industry environments have shown that some phages can maintain viable counts of 10^2^–10^3^ PFU/mL even after routine disinfection procedures [219], providing robust evidence for their stability in practical applications.

Research also reveals that during the final stage of the phage lytic cycle, the lysis of host bacteria not only releases progeny phage particles but also liberates fragments of host genomic DNA into the surrounding environment [220]. If these DNA fragments carry ARGs, they can potentially be incorporated into competent bacteria through natural transformation. This finding highlights a critical consideration for refining hospital infection control strategies. When developing phages for environmental decontamination or clinical therapy, it is essential to select strictly lytic phages that are confirmed to be free of ARGs and virulence genes. Future efforts should focus on optimizing phage formulations, application protocols, and standardized operating procedures, while also ensuring clinical convenience and cost effectiveness.

### 3.3 Prevention and Control of Waterborne Pathogens in Hospitals

Hospital water systems, including sinks, faucets, and wastewater drainage systems, serve as critical reservoirs for MDROs and are significant contributors to healthcare‐associated infection outbreaks [221, 222]. The consistently moist and oxygenated environment of these fixtures provides optimal conditions for microbial survival and biofilm formation, facilitating the long‐term colonization of nonfermenting bacteria such as *P. aeruginosa* and *A. baumannii*. The efficacy of routine disinfection measures against water systems is notably limited [223]. A randomized controlled trial (RCT) conducted across 26 clinical wards demonstrated that even with weekly disinfection using sodium hypochlorite or thermal steam treatment over four weeks, the elimination rate of MDROs remained inadequate [224]. Research confirms that biofilms in sink traps grow at a rate of approximately one inch per day within pipes. Additionally, surfaces can be recolonized within a week after sodium hypochlorite disinfection [225, 226], leading to the dissemination of pathogens as contaminated droplets through water flow to surrounding areas.

With the increasing severity of hospital waterborne contamination, phages present a promising novel approach for the biocontrol of hospital water systems [227]. Zapata‐Montoya et al. validated the bactericidal efficacy of phages in simulated wastewater, targeting CRKP CG258 strains. Phages FKP3 and FKP14 achieved a 99% reduction over a 54‐h intervention period, while an optimized phage combination reached 99.99% clearance efficiency within 36 h [228]. Sadeqi et al. found that a phage cocktail applied to hospital wastewater led to near‐complete elimination of *A. baumannii* and *S. maltophilia*, with other common hospital pathogens, including *E. coli*, *P. aeruginosa*, and *S. aureus*, also exhibiting significant reductions. Importantly, they noted that phages retained high biological activity in the complex, real‐world wastewater environment [229]. Furthermore, phages leverage their high host specificity to target and remove specific pathogens or dysfunctional bacterial populations that contribute to common wastewater treatment issues, such as sludge bulking, foaming, and excessive biofilm growth. This targeted approach helps preserve the activity of essential functional microbial communities, including nitrifying and polyphosphate‐accumulating bacteria. Several virulent phages, including T7, SPI1, GTE7, and PhaxI, have been identified, and their effectiveness in clearing biofilms and restoring sludge settleability has been validated at both laboratory and pilot scales [230]. Phages counter AMR spread through a dual mechanism: reducing the host bacterial population, which can carry MGEs, and through phage‐encoded depolymerases that degrade the biofilm matrix, disrupting the microenvironment that facilitates HGT of resistance determinants.

Phages can also function as effective biological tracers for environmental monitoring. Studies have shown that genetically engineered T7 phages, conjugated with magnetic particles, significantly enhance detection sensitivity for *E. coli* to 100 CFU/mL, reducing detection time from several days to just a few hours [231, 232]. Additionally, the dynamic changes within phage populations can serve as reliable indicators of the growth and decline patterns of their host bacteria, offering a novel technical pathway for real‐time performance assessment of wastewater treatment systems [233].

However, challenges remain regarding the practical application of phages. Phage genomes may carry ARGs as well as auxiliary metabolic genes involved in carbon, nitrogen, and sulfur cycles [234, 235, 236]. Therefore, safety assessments at the whole‐genome level are critical during phage screening and application. Moreover, bacteria can develop resistance to phage infection through various mechanisms, such as surface receptor modification and defense systems [237]. This coevolutionary dynamic between host and phage may influence the long‐term stability and efficacy of phages in engineered applications. Consequently, ongoing monitoring of bacterial population shifts is essential, and rational design and continuous optimization of phage cocktail formulations must be prioritized [238, 239]. Despite these challenges, data from both laboratory and real‐world settings consistently demonstrate that phages hold considerable potential in hospital wastewater treatment.

## 4 Phage‐Based Vaccine Development

**FIGURE 3:**
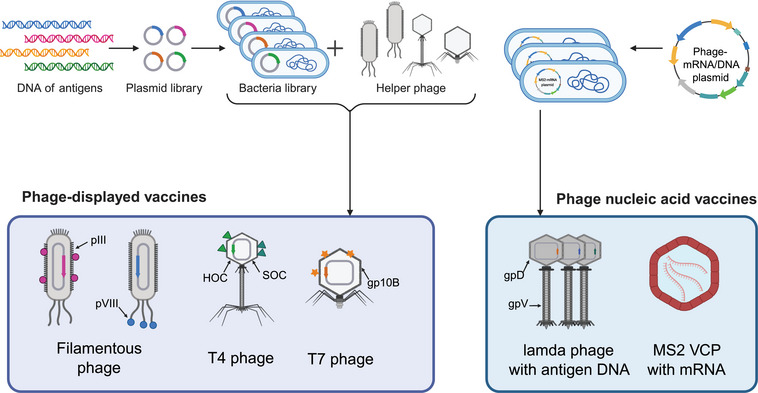
Phage‐based vaccines. The left frame presents phage‐displayed vaccines with antigens on surface capsid proteins. The right frame indicates phage nucleic acid vaccines encapsulating antigen‐encoding DNA or mRNA sequences within the viral capsid.

Traditional vaccines are typically prepared by inactivating or attenuating whole pathogenic microorganisms, thereby eliminating their toxicity and pathogenicity while retaining their immunogenicity [240]. In contrast, phages, which are viruses that specifically lyse bacterial cells and are not considered true human pathogens, present a unique alternative. As such, phages are increasingly recognized as versatile platforms for vaccine design and delivery, particularly for protein subunit and nucleic acid vaccines. This opens new possibilities for the development of novel phage‐based vaccine strategies. Current research on phage‐based vaccines primarily focuses on two approaches: phage‐displayed vaccines and phage nucleic acid vaccines (Figure 3).

### 4.1 Phage Display‑Based Vaccines

Phage‐displayed vaccines are based on phage display technology, first introduced by Smith et al. in 1985[241]. There are two primary strategies for displaying antigens on phage surfaces. One approach involves inserting the gene encoding the antigen near the gene for a phage capsid protein, enabling the phage to express and display the antigen directly. The second strategy uses a phage‐displayed antigen‐binding peptide to capture and present the target antigen. However, due to the low efficiency of the capture and amplification steps in the latter method, genetic engineering has become the most commonly used strategy for constructing phage‐displayed vaccines [242].

Filamentous phages, such as M13 and fd, are the most commonly used systems for phage display. Antigenic peptides are typically fused to and displayed on the phage's protein pVIII or pIII. pVIII, the major coat protein, has a high density of antigen display sites, but is generally limited to presenting short peptides of fewer than 20 amino acids [243]. pIII, located at the phage tip, is a minor coat protein that can accommodate larger proteins up to 100 kDa. However, the number of pIII display sites is limited, which can result in a lower overall immunogenicity of the resulting vaccine [244]. Unlike typical lytic phages, the coat proteins of filamentous phages are synthesized and secreted across the inner membrane into the periplasm, where assembly occurs before the phage is extruded from the cell [245, 246]. This nonlytic release mechanism partially prevents contamination of the vaccine preparation with bacterial components. However, the displayed antigenic proteins must be structurally stable enough to withstand the challenges associated with the assembly and secretion process. Early research demonstrated that displaying a hepatitis B virus epitope on the surface of a filamentous phage could induce a specific cytotoxic T lymphocyte response in mice without the need for adjuvants [247]. More recently, Wang et al. employed nanoengineering to modify the M13 phage surface with manganese dioxide particles. In the acidic intracellular environment, Mn^2+^ ions are released, promoting phage escape from endosomes into the cytoplasm and enhancing antigen expression. Simultaneously, this process stimulates innate immunity by activating the cGAS–STING pathway [248]. This composite platform successfully elicited both cellular and humoral immune responses in a mouse influenza model, offering significant cross‐protective efficacy.

Lytic phages T4 and T7 are also widely used in phage display applications. The T4 phage features a dual display system, enabling multiple antigens to be presented simultaneously on its highly antigenic outer capsid and small outer capsid proteins. This capability can trigger robust, high‐level immune responses [249, 250]. The T7 phage platform offers advantages such as rapid amplification, outpacing filamentous and lambda phages in replication speed, and the ability to accommodate relatively large antigen inserts of up to 2 kb [251]. Zhu et al. successfully displayed multiple variants of the influenza A virus matrix protein 2 ectodomain (M2e) and the hemagglutinin stalk domain on the T4 capsid surface [252]. Similarly, the T7 phage platform has been utilized to display the M2e antigen, generating antibodies that recognized native M2 protein on infected cells, which conferred cross‐protection against H1N1 and H3N2 viral challenges in mouse models [253]. The Tao team developed an adjuvant‐free bivalent anthrax‐plague vaccine using an in vitro assembly process to densely display the anthrax protective antigen (PA) and an engineered plague F1–V fusion antigen on the T4 capsid surface. In mouse, rat, and rabbit models, this vaccine induced potent anthrax‐ and plague‐specific humoral and cellular immunity, eliciting a balanced Th1/Th2 response. Notably, it provided complete protection against simultaneous challenges with lethal doses of anthrax spores and Yersinia pestis [254]. Another study demonstrated that intranasal administration of a T4 phage vaccine displaying an engineered Spike protein stimulated broad‐spectrum neutralizing antibodies and Th1‐biased T cells. Additionally, it induced high levels of secretory IgA and tissue‐resident memory T cells in the respiratory mucosa, achieving “sterilizing immunity” and complete protection against multiple SARS‐CoV‐2 variants, including Delta [255].

For established pathogens, phage display technology enables the precise construction of prophylactic vaccines. In response to novel or emerging pathogens, the extensive random peptide libraries based on phage display can provide a broad range of antigenic epitopes, offering critical target information for vaccine design. The application of phage‐displayed vaccines has expanded beyond traditional infectious diseases, progressively moving into challenging therapeutic areas such as oncology. Yue et al. displayed two distinct peptides on the pIII and pVIII coat proteins of the fd filamentous phage, one targeting melanoma (IP) and the other blocking the PD‐1/PD‐L1 interaction (HH). This led to the creation of the bifunctional phage fd–HH–IP. Upon intravenous injection in mice, the engineered phage actively targeted and accumulated in tumor tissues. The high local density of HH peptides effectively blocked the PD‐1/PD‐L1 pathway in situ, achieving a potent localized blocking effect. This strategy demonstrated superior tumor treatment efficacy compared with traditional molecular inhibitors [256]. In another study, an M13 phage‐based nanovaccine, when combined with anti‐PD‐1 therapy, achieved a complete response rate of 75% in a colon cancer model. This treatment also induced durable immune memory lasting up to 1 year, providing 100% protection against tumor rechallenge in the animals [257]. The phage platform offers low‐cost, large‐scale production and high antigen‐loading capacity, marking a shift in phage display technology from a powerful in vitro screening tool to a programmable therapeutic platform. This platform integrates antigen discovery, targeted delivery, and immune modulation, positioning it as a promising approach for both infectious disease and cancer immunotherapy.

### 4.2 Phage Nucleic Acid Vaccines

Among phage‐based nucleic acid vaccines, DNA vaccines have garnered significant attention due to their straightforward production and good stability. However, their clinical application in humans has long been impeded by challenges in delivery efficiency and immunogenicity. While mRNA vaccines have made breakthroughs in these areas with the aid of lipid nanoparticles (LNPs) and adjuvants, they face hurdles such as complex manufacturing processes, high costs, and stringent cold‐chain requirements [258].

The lambda phage, with its large genome, can encapsulate a variety of genes encoding foreign antigens, which can be displayed on nonessential proteins such as the gpD head protein or gpV tail protein [259]. However, its lysogenic lifestyle results in lower titers during large‐scale production compared with filamentous phages [249]. In pioneering work, Hashemi et al. inserted expression cassettes containing a CMV promoter and genes for herpes simplex virus‐1 glycoprotein D [260], hepatitis B virus surface antigen [261], or human papillomavirus‐16 E7 protein [262] into the lambda phage genome. The recombinant phage particle, once purified, was directly injected as a vaccine. Although the antigen gene copy number delivered by the phage was lower than that of plasmid DNA at the same dose, it still elicited a potent immune response, suggesting high delivery or immunostimulatory efficiency. Notably, the antibody response was comparable to or even superior to that induced by commercially available recombinant protein vaccines [263]. Notably, while the host generated high‐titer antibodies against the phage, these did not suppress subsequent immune responses to the vaccine antigen [261]. More recently, Xu et al. investigated the feasibility of using the T7 phage as a DNA vaccine carrier. They found that during infection of *E. coli*, T7 phages could specifically recognize and encapsulate DNA plasmids containing a particular “anchoring sequence” (AS2) with an efficiency exceeding 95%. However, the immunogenic efficacy of this system in animal models has yet to be evaluated [264].

A novel mRNA delivery platform based on the MS2 capsid protein (VCP) was recently developed by Su et al. This system bypasses the in vitro transcription and LNP encapsulation processes typical of conventional mRNA vaccine production. Instead, it employs a “one‐step” manufacturing strategy that simultaneously synthesizes and encapsulates both the capsid protein and the target mRNA, resulting in structurally uniform virus‐like particles. This platform efficiently encapsulates and protects the mRNA from degradation for up to 3 months when stored at 4°C. Additionally, both cellular and mouse models confirmed the effective cytoplasmic delivery and expression of the mRNA. The expressed antigen successfully induced a specific CD8^+^ T cell response and demonstrated measurable tumor growth inhibition [265].

Most traditional vaccines primarily rely on protein‐based antigens, which typically induce minimal cellular immunity. In contrast, phages can simultaneously trigger both humoral and cellular immune responses. Langbeheim et al. demonstrated that phages activate cellular immunity, with whole phage particles exhibiting stronger immunostimulatory capacity than isolated phage coat proteins [241, 266]. The CD4^+^ and CD8^+^ T cells generated through this response play pivotal roles in controlling viral infections and eliminating tumor cells. However, a significant challenge for phage‐based vaccines lies in the humoral immune response. Antibodies targeting the phage can bind to its tail structures, preventing infection and reducing the vaccine's effectiveness [267]. Additionally, the interaction between antibodies and phages often impedes their activity and immunogenicity, posing a key barrier in the development of effective phage‐based vaccines.

Phages offer several advantages for vaccine development, including low production costs, scalability, and stability under harsh conditions, which facilitates both storage and transportation. Nonetheless, significant challenges remain in translating laboratory research into clinically viable vaccine products. This transition requires thorough evaluation of phage safety and their in vivo biological behavior. Moreover, the precise mechanisms by which phage‐based vaccines elicit immune responses in the body are not yet fully understood.

## 5 From Laboratory to Clinical: Progress In R&D and Trials

Phage therapy has evolved from initial laboratory studies to clinical applications, progressing through stages such as in vitro screening, animal model validation, and both preclinical and clinical trials. This section reviews key advancements in phage therapy, examining animal models, preclinical research, clinical trials, as well as regulatory and ethical considerations. A comprehensive summary of these studies is provided in Table 3, offering a detailed analysis of the major technical and regulatory challenges and exploring future directions for development. This review aims to provide both theoretical insights and practical references for the clinical implementation of phage therapy.

**TABLE 3:** Preclinical and clinical studies of phage therapy.

Disease	Pathogen	Treatment	Outcome	Adverse reaction	Phase	References or ClinicalTrials.gov ID
Bacteremia	*S. aureus*	AB‐SA01 1 × 10^9^ PFU/mL twice daily for 14 days	62% clinical improvement, 38% mortality within 28 days	None	Case report	[269]
	*S. aureus*	AP‐SA02 intravenous drip	Not mentioned	Not mentioned	Ib/IIa	NCT05184764
Cystic fibrosis	*P. aeruginosa*	Bronchoscopic instillation + nebulization twice daily	Clinical improvement; FEV1% increased	None	Case report	[270]
	*P. aeruginosa*	AB‐PA01 intravenous injection (every 6 h) 4 × 10^9^ PFU per dose for 8 weeks	No recurrence of *PA* pneumonia	None	Case report	[285]
	*P. aeruginosa*	Bronchoscopic instillation + nebulization twice daily	Not mentioned	Not mentioned	I/II	[278]
	*P. aeruginosa*	3 mL YPT‐01 nebulization for 7 days, once daily	Decreased bacterial count in sputum culture and improved pulmonary function	None	II	NCT04684641
	*P. aeruginosa*	Aerosol inhalation of AP‐PA02	Decreased bacterial count in sputum culture and improved pulmonary function	None	Ib/IIa	NCT04596319
	*P. aeruginosa*	Intravenous injection of 10^7–9^ PFU bacteriophage therapy	Not mentioned	Not mentioned	Ib/II	NCT05453578
Acute pulmonary infection	*K. pneumoniae*	Bacteriophage: 10^7^ PFU (intranasal administration); Cefotaxime: 150 mg/kg (intraperitoneal injection, every 12 h)	Improvement in pulmonary pathology and reduction in bacterial load	None	Animal experimentation	[271]
	*P. aeruginosa*	Intratracheal administration at 2.5 × 10^9^ PFU, combined with meropenem	100% survival rate, improved lung pathology, and reduced bacterial load	None	Animal experimentation	[272]
	*K. pneumoniae*/ *P. aeruginosa*	Nebulized inhalation twice daily, with a total titer of 6–9 × 10^9^ PFU/mL per dose, for 8 weeks	Reduced bacterial load	None	Case report	[286]
Acute osteomyelitis	*S. aureus*	Intravenous injection at a dose of 40 mg/kg Daptomycin: intraperitoneal injection every 12 h at a dose of 60 mg/kg for 4 days	Significant therapeutic effect	None	Animal experimentation	[274]
	*K. pneumoniae*	Local administration	Significant therapeutic effect	None	Case report	[281]
Prosthetic joint infection	*S. aureus*/*P. aeruginosa*	Local administration	Reduced bacterial load	None	Animal experimentation	[290]
	*S. aureus*	Local administration + intravenous administration	Complete clearance of infection	Mild elevation of transaminases	Case report	[280]
	*S. aureus*	Local administration	Complete clearance of infection	None	Case report	[290]
	*P. aeruginosa*	Local administration	Complete clearance of infection	None	Case report	[290]
	*S. aureus*	Local administration + intravenous administration	Not mentioned	Not mentioned	I	NCT06827041
	*S. aureus*	Local administration + intravenous administration	Not mentioned	Not mentioned	I/II	NCT06456424
	*Morganella morganii*	Local administration + intravenous administration	Not mentioned	Not mentioned	I/II	NCT06814756
Chronic bacterial prostatitis	*S. aureus*, etc.	Oral, rectal suppositories, and urethral instillation	No pathogenic bacteria growth, symptoms completely resolved	None	Case report	[287]
	*E. coli*/*S. aureus*	Oral, rectal suppositories, and urethral instillation	Bacterial load reduction	None	Case report	[288]
Diabetic foot	*S. aureus*	Local administration	Cured, no recurrence	None	Case report	[290]
	*S. aureus*/*P. aeruginosa*	Local administration	Reduced bacterial load with high wound closure rate	None	I/IIa	NCT04803708
	*S. aureus*	Local administration	Not mentioned	Not mentioned	I/II	NCT04289948
	*S. aureus*	Local administration	Not mentioned	Not mentioned	IIb	NCT05177107
Urinary tract infection	*E. coli*	Intravesical instillation	bacterial load reduction	None	I	NCT04191148
	*E. coli*	Intravesical instillation	Not mentioned	Not mentioned	I/II	NCT04287478
	*E. coli*/*P. aeruginosa*, etc.	Intravesical instillation	Not mentioned	Not mentioned	II/III	NCT03140085
	Enterobacteria	Intravesical instillation	Not mentioned	Not mentioned	III	NCT05967130
Crohn's disease	*E. coli*	Single treatment: 1.4 × 10^8^ PFU, long‐term treatment: twice daily, 2 × 10^9^ each time, for 15 consecutive days	The effect of single treatment is limited; long‐term treatment shows significant efficacy.	None	Animal experimentation	[277]
Typhoid fever	*Salmonella typhi*	Acute infection treatment: intraperitoneal injection; chronic carrier state treatment: oral administration	Complete eradication of infection	Mild hemolysis and inflammatory response, with no severe adverse reactions	Animal experimentation	[279]
Acute tonsillitis	*S. aureus*, etc.	Inhale 5 mL of PCL via nebulization every 5 days	Not mentioned	Not mentioned	III	NCT04682964

### 5.1 Preclinical Research Phase: Validation of Animal Models

#### 5.1.1 Research Objectives and Significance

The validation of phage safety in vivo is a critical prerequisite for clinical translation. Numerous studies have shown that systemic phage administration does not induce significant toxicity or excessive immune responses in various animal models. For example, oral administration of high‐dose phages in Charles Foster rats did not result in immune abnormalities or tissue toxicity [268]. Similarly, intravenous phage therapy in Australian hospital patients with severe *S. aureus* infections did not cause acute adverse reactions [269]. Phages administered via bronchoscopy and nebulization also demonstrated good tolerability in cystic fibrosis patients [270], highlighting the safety and versatility of different delivery routes.

Additionally, PK/PD studies are essential for understanding phage distribution, metabolism, clearance patterns, and bactericidal efficacy over time. Through animal models, researchers can assess phage half‐life, dose–response relationships, and optimize dosing intervals. For instance, in a murine lung infection model, the combination of phages and cephalosporins significantly reduced bacterial load and prolonged survival, demonstrating synergistic pharmacodynamic effects [271]. These pharmacokinetic insights are essential for informing clinical dose design.

Exploring different administration routes is also key to optimizing therapeutic efficacy. In animal models, direct airway administration achieves high local concentrations rapidly, enhancing the therapeutic effect. In contrast, systemic routes such as intraperitoneal injection are more suitable for treating systemic infections. Local delivery methods, such as intratracheal instillation, have been shown to effectively clear MDR *P. aeruginosa* lung infections [272], and systemic administration combined with antibiotics also exhibits synergistic benefits [272].

Finally, the combination of phages and conventional antibiotics presents a promising strategy to combat antibiotic resistance. Phages can augment the bactericidal effects of antibiotics and reduce the emergence of resistant strains. Multiple animal studies have confirmed that phage–antibiotic combination therapy leads to greater bacterial load reduction and improved infection outcomes compared with monotherapy [271, 273]. This synergistic effect is evident not only in acute infections but also in chronic and biofilm‐associated infection models.

In conclusion, preclinical studies have comprehensively validated the safety, PK, PD, administration routes, and synergistic effects of phage therapy with antibiotics through animal models. These findings provide a strong foundation for the design and execution of future clinical trials, offering significant scientific and clinical value.

#### 5.1.2 Key Animal Models and Major Findings

In preclinical phage studies, the selection of animal models is closely aligned with infection types and clinical needs, primarily encompassing acute infection models, chronic and biofilm‐associated infection models, and immunocompromised host models.

Acute infection models, such as those simulating sepsis and pneumonia, replicate clinically critical infection states to assess the rapid bactericidal effects and survival benefits of phages. For instance, in a mouse model of acute *P. aeruginosa* lung infection, the combination of phages and antibiotics significantly improved survival rates and reduced bacterial load in the lungs [271, 272]. Similarly, in a rat model of *S. aureus* osteomyelitis, phage treatment markedly decreased bacterial counts and alleviated bone tissue pathology [274]. In both porcine and human in vitro burn models, phage therapy demonstrated superior efficacy compared with conventional antibiotics in treating burn infections [78], highlighting the potential of phages in managing acute infections.

Chronic and biofilm‐associated infection models, including catheter‐related infections, chronic pulmonary infections, and osteomyelitis, focus on evaluating the ability of phages to penetrate biofilms and eliminate persistent bacterial populations. Bacteria within biofilms exhibit heightened antibiotic resistance, but phages can effectively infiltrate and lyse these bacteria. Numerous in vitro and in vivo studies have demonstrated that phages significantly inhibit and eradicate biofilm formation. For example, treatment with phages on stainless steel surfaces contaminated with *E. coli* biofilms resulted in a reduction of bacterial counts by over 6 log [275]. In mouse models of cystic fibrosis, phage therapy decreased bacterial load in the respiratory tract and mitigated inflammation [276, 277]. In models of chronic osteomyelitis and wound infections, phages effectively reduced biofilm‐associated bacterial counts and facilitated tissue repair [278, 279].

Immunocompromised host models are employed to assess the safety and efficacy of phages in immunodeficient individuals. Given that immunosuppressed patients often present with complex infections, animal models such as immunosuppressed mice or rabbits are used to simulate clinical immunocompromised states. Research indicates that phage therapy remains effective in managing infections in these models, with no significant toxic side effects observed in immunodeficient animals [280]. Additionally, these models help investigate the interaction between phages and the immune system, revealing the potential role of phages in immune modulation [281].

Overall, studies using animal models consistently demonstrate that phages, particularly multistrain cocktails, exhibit favorable safety profiles across most infection models, significantly reducing bacterial load and improving pathological outcomes. The cocktail approach enhances efficacy and mitigates resistance development by targeting multiple bacterial strains and resistance mechanisms [75, 282]. These findings provide a strong scientific foundation for advancing phage therapy into clinical research, supporting its development as a promising treatment for antibiotic‐resistant infections.

### 5.2 Clinical Research Phase: Human Trial Evidence

#### 5.2.1 Case Reports and Case Series

In the early stages of clinical phage therapy research, case reports primarily focused on patients with MDR bacterial infections, including those with cystic fibrosis, burn injuries, or prosthetic joint infections. These reports highlighted the successful application of phage therapy as part of personalized treatment or compassionate use. For example, Law et al. [283] documented the case of a 26‐year‐old cystic fibrosis patient awaiting lung transplantation, who developed MDR *P. aeruginosa* pneumonia and persistent respiratory failure during the waiting period. After 8 weeks of treatment with intravenous AB‐PA01 (a cocktail of four lytic phages produced by AmpliPhi Biosciences, USA) at a dosage of 4 × 10^9^ PFU/mL, 5 mL, the patient experienced a reduction in supplemental oxygen usage and sputum volume, resulting in significant symptomatic improvement. No recurrence of *P. aeruginosa* pneumonia or cystic fibrosis exacerbation occurred within 100 days posttreatment, and 9 months later, the patient successfully underwent bilateral lung transplantation.

Over the past 5 years, numerous case reports have demonstrated the potential of phage therapy as a salvage treatment for MDR infections, particularly in chronic, refractory conditions such as pulmonary infections and chronic bacterial prostatitis (CBP).

In a pulmonary infection case involving carbapenem‐resistant *P. aeruginosa*, an 8‐week course of nebulized phage therapy was administered [284]. Posttreatment, the patient showed reduced bacterial load in sputum, improved systemic inflammatory markers, and significant resolution of pulmonary effusion. Although phage‐resistant bacterial strains were isolated during treatment, whole‐genome sequencing and infection model analysis revealed attenuated virulence in these resistant strains, demonstrating a “virulence‐fitness” trade‐off effect that ultimately led to a positive clinical outcome for the patient [284].

In genitourinary infections, case reports on CBP are particularly notable. Due to the complex anatomical structure and the tendency of bacteria to form biofilms, conventional antibiotic treatments for CBP are often ineffective [285, 286]. One case involved a CBP patient infected with multiple pathogens, including MRSA, *Staphylococcus haemolyticus*, and *Enterococcus faecalis*, who received phage therapy from the Eliava Institute after several unsuccessful antibiotic treatments [285]. Following therapy, the patient showed significant improvement in symptoms and a reduction in bacterial load [285]. Another CBP patient with an MDR *E. coli* infection underwent two courses of phage therapy after 5 years of failed antibiotic treatment, ultimately achieving long‐term symptom relief and pathogen clearance [273].

These cases highlight the promising potential and safety of phage therapy for chronic infections caused by MDR bacteria that are resistant to conventional antibiotics. Notably, the emergence of phage‐resistant bacteria during treatment does not necessarily imply clinical failure, as resistance development can sometimes be accompanied by a reduction in bacterial virulence.

However, case reports and series have inherent limitations. They lack control groups and provide low‐quality evidence, making it difficult to exclude potential confounding factors, thus precluding statistical confirmation of phage therapy efficacy [287]. Additionally, the small number of cases limits their representativeness of a broader patient population and pathogen diversity, restricting the generalizability of the results. The heterogeneity of individualized treatments further complicates efficacy evaluation and presents challenges in establishing standardized treatment protocols. While case reports provide preliminary clinical support for phage therapy, larger, well‐designed clinical trials are necessary to validate its safety and efficacy and to establish standardized guidelines for its application [288].

#### 5.2.2 Progress of Clinical Trials

Current studies on phage therapy registered on ClinicalTrials.gov primarily target diseases such as cystic fibrosis, chronic lung diseases, MDR infections caused by *P. aeruginosa*, gastrointestinal infections, prosthetic joint infections, diabetic wound infections, and MDR bacterial UTI. While phage therapy is still in its early stages, further optimization of PK/PD models is essential [287].

Completed Phase I clinical trials have explored various routes of administration, including intravenous injection, inhalation, and topical application, focusing on evaluating the tolerability and pharmacokinetic properties of phage preparations in healthy volunteers and patients. The findings generally indicate that phage therapy is well tolerated, with no serious adverse events reported, supporting its clinical safety [289]. Preliminary data have also provided insights into the clearance half‐life, distribution, and metabolic pathways of phages in the human body, which inform the design of subsequent treatment regimens and dose optimization. For instance, one study found that the half‐life of phages in the bloodstream was several hours following intravenous administration, suggesting that adjustments in dosing frequency may be necessary to maintain effective concentrations. PK/PD studies on different routes of administration offer valuable scientific data for clinical dose setting and treatment protocols [290].

Phase II clinical trials shift the focus to exploring the efficacy of phage therapy for various infections, including *P. aeruginosa* infections in cystic fibrosis patients, diabetic foot ulcers, chronic otitis media, UTI, and burn wound infections. Initial results showed positive outcomes in bacterial clearance, wound healing, and symptom improvement in some patients [291]. However, therapeutic effects varied considerably, with limited responses observed in certain cases, highlighting the importance of strain matching. The precise binding between phages and pathogenic bacteria is critical to efficacy, with the effective matching of phage strains being decisive. Additionally, optimizing dosage, administration frequency, and treatment duration significantly impacts therapeutic outcomes. Phase II trial results offer essential guidance for designing Phase III trials, particularly in establishing inclusion criteria and efficacy evaluation metrics [292].

Currently, the number of Phase III clinical trials remains limited, posing a significant bottleneck in phage therapy development. A few Phase III trials targeting conditions like MDR *P. aeruginosa* pulmonary infections have either been initiated or are in preparation [289]. These trials primarily use randomized, double‐blind, placebo‐controlled designs, with some incorporating antibiotic combination therapy to objectively assess phage therapy's efficacy and safety. The design of Phase III trials aims to enhance the level of evidence, address the heterogeneity seen in earlier trials, and strengthen the statistical foundation for therapeutic efficacy. As these trials progress, Phase III results will provide critical support for the standardized promotion of phage therapy and the approval of related drugs [287].

#### 5.2.3 Key Challenges and Lessons Learned

Phage therapy faces several challenges in clinical trials. One major issue is the heterogeneity among patients and pathogens, which complicates the evaluation of efficacy. Variations in immune status, infection sites, and bacterial strain diversity result in significant differences in treatment responses, making efficacy analysis complex. Additionally, the intricate interactions between phages, hosts, and bacteria pose further challenges in interpreting results. Host immune responses can influence phage activity and persistence, while bacteria may develop resistance through mutations, reducing efficacy.

Another obstacle is the lack of standardized dosing regimens. Further systematic research is required to establish optimal dosages, frequencies, treatment durations, and administration routes [292]. These challenges highlight the importance of precise strain matching, personalized dosing, and comprehensive immunomodulation strategies to improve therapeutic outcomes. Future efforts should focus on integrating basic research with clinical trials, optimizing phage preparations and treatment protocols, and enhancing the feasibility and effectiveness of clinical applications [293].

### 5.3 Regulatory and Ethical Oversight Framework

#### 5.3.1 Global Regulatory Landscape and Challenges

Regulatory systems for phage therapy differ across countries and regions, though they share certain similarities. In the United States, the US FDA has established an approval pathway for phage therapies that includes specific production and clinical development requirements to facilitate market authorization [294]. Therapeutic phages are classified as biologics or drugs, requiring compliance with GMP standards and the submission of comprehensive pharmaceutical, nonclinical, and clinical data to demonstrate safety and efficacy. While this regulatory approach ensures quality control and standardization in clinical applications, it also presents challenges.

The biological characteristics of natural phages are complex and highly variable, particularly when it comes to multicomponent phage cocktails. The combinatorial diversity among different phages complicates quality control and batch‐to‐batch consistency, creating regulatory hurdles. Moreover, regulatory frameworks for overseeing “personalized” phage preparations, such as hospital formulations, are still underdeveloped. Balancing individualized treatment needs with standardized product regulation is a significant challenge. As precision medicine advances, there is an increasing demand for phage matching based on rapid diagnostic results. This has led regulatory agencies to consider rapid diagnostic‐based approval pathways or adaptive regulatory approaches to expedite patient access to treatment. However, the mechanisms for evaluating safety and efficacy within these frameworks are still being refined.

Currently, the European drug regulatory system exhibits significant deficiencies in ethical oversight. For instance, Belgium has implemented strategies like the “Magistral Phage” preparation to address these regulatory gaps [295]. The Middle East, on the other hand, is still in the early stages of developing regulatory frameworks for phage therapy, requiring further research and refinement to fully unlock its potential [296]. Although the new European Clinical Trials Regulation strengthens safety measures and protects trial participants’ rights, the role of ethics committees is largely limited to pretrial approval, lacking ongoing oversight. Moreover, insufficient communication and transparency between ethical review bodies and regulatory agencies have led to fragmented supervision [297]. Globally, ethical oversight faces challenges such as inadequate legal frameworks, poorly structured committees, limited resources, and a lack of accountability. This highlights the need to strengthen ethical governance systems tailored to local contexts [298].

Emerging technologies, such as artificial intelligence, also face complexities in regulatory oversight in the medical field, with no globally coordinated regulatory frameworks in place. Similarly, emerging biotechnologies like phage therapy encounter challenges, including algorithmic bias and unclear ethical responsibilities, necessitating more adaptive regulatory models [299, 300]. In summary, global regulation of phage therapy is rapidly evolving, requiring a balance between innovation, flexibility, and ensuring safety and efficacy. This approach will help foster coordination and refinement of regulatory systems.

#### 5.3.2 Ethical Considerations

The ethical oversight system for phage therapy faces numerous challenges, particularly regarding the ethical principles and conditions surrounding compassionate use and expanded access. As an experimental and personalized therapeutic product, obtaining informed consent from patients and their families is crucial. This process must clearly communicate the experimental nature of the product, the personalized components involved, and potential risks, such as gene transfer [297].

Right‐to‐try legislation, which empowers patients to access experimental therapies outside traditional approval systems, aligns with the personalized, rapid‐matching characteristics of phage therapy, potentially facilitating its application. However, this raises ethical concerns that necessitate rigorous risk assessments and protective measures for patients [301]. Furthermore, ensuring equitable access to personalized and potentially costly phage therapy for a broad patient population presents a critical issue in ethical oversight. This includes concerns about resource allocation, accessibility, and social justice [297].

While the environmental risks associated with phage therapy are relatively low, they still require thorough evaluation and monitoring. Introducing phages into the environment could have ecological consequences and raise liability concerns, making it essential for ethical oversight to include considerations of environmental safety and social responsibility, ensuring the sustainable development of phage therapy. In terms of ethical governance, China has recently strengthened its framework for medical research, emphasizing a principle of “ethics first” and advocating for proactive, preventive governance through open collaboration. This framework could serve as a model for regulating emerging technologies like phage therapy [302].

In conclusion, the ethical regulation of phage therapy must balance technological innovation with the protection of patient rights, enhance informed consent procedures, and address issues related to equitable access and environmental safety. The goal is to create a comprehensive, dynamic, and adaptable ethical oversight framework that supports the safe, effective, and equitable clinical application of phage therapy.

## 6 Future Perspectives and Conclusion

Phage therapy has progressed from a conventional antibacterial approach to a multifaceted therapeutic strategy, encompassing direct pathogen elimination, prevention of nosocomial infections, and immune modulation. The synergistic use of antibiotics and phages, advancements in biomaterial‐enhanced delivery systems, and genetic engineering have broadened host specificity and improved lytic efficiency. As eco‐friendly biocontrol agents, phages effectively disrupt the transmission of drug‐resistant bacteria within hospital environments. Additionally, leveraging their superior antigen display and nucleic acid delivery capabilities, phages are increasingly being explored as novel vaccine platforms, providing innovative solutions for antiviral and cancer immunotherapy. However, the complexity of phages as “living drugs” presents several challenges in modern medicine, including clinical application, industrial production, and regulatory oversight.

Currently, phages are classified as medicinal products only in countries such as Georgia, Russia, and Poland, where they hold registration licenses issued by national drug regulatory agencies. In these regions, hospitals and pharmacies offer various phage preparations. However, these products are not manufactured in compliance with modern GMP standards and have not undergone standardized clinical trials. As a result, they have not received approval from regulatory authorities in other countries [303]. In China, the United States, Canada, the European Union, and the United Kingdom, phages are still classified as “biological medicinal products” or “investigational drugs,” with no phage products having gained formal marketing authorization in these jurisdictions [12].

Clinical phage therapy employs two main strategies. The first, fixed‐composition phage therapy, uses preformulated phage cocktails with defined components, primarily targeting infections caused by *S. aureus* and *P. aeruginosa*. One such example is the ongoing PHAGE trial, a Phase 1b/2 randomized controlled study funded by the Antibacterial Resistance Leadership Group of the National Institute of Allergy and Infectious Diseases [304]. The development of these fixed phage preparations follows a pathway similar to traditional pharmaceuticals, allowing for large‐scale, standardized production. Regulatory agencies, such as the US FDA and EMA, are working to integrate these products into existing biologics approval pathways, with requirements including the establishment of phage and host cell banks that comply with International Council for Harmonisation standards [305]. Manufacturing processes must adhere to GMP standards to ensure purity, consistent potency, and minimal endotoxin levels. Additionally, comprehensive PK/PD data are essential, along with well‐designed RCTs to demonstrate the superiority of phage therapy over the best available antibiotic treatment, typically in combination. However, strict inclusion criteria for RCTs often exclude patients who have already received antibiotic treatment, presenting challenges in recruiting eligible subjects [306]. Furthermore, a preformulated, “broad‐spectrum” phage product often fails to address the genetic diversity of bacterial strains encountered in real‐world patient infections. Notably, only two out of seven such trials have achieved therapeutic success [307], highlighting the limitations of a “one‐size‐fits‐all” approach.

The second strategy, personalized treatment, involves selecting or preadapting therapeutic phage combinations from a characterized phage bank tailored to the patient's specific pathogen. This approach is often more feasible in clinical practice and has shown a higher success rate. A multinational, multicenter retrospective observational study conducted by Queen Astrid Military Hospital, involving 100 cases of difficult‐to‐treat infections, suggested a higher likelihood of infection eradication when phage therapy was combined with antibiotics [42]. However, each treatment is unique, leading to considerable variability in the composition, dosage, and duration of the phage product used. Furthermore, production standards are typically set by research institutions internally, rather than being strictly controlled under GMP standards [308, 309]. As a result, the personalized approach does not provide the systematic efficacy and safety data from a homogeneous patient population required for traditional drug marketing authorization. Consequently, it remains largely in a regulatory “gray zone” under compassionate use or special authorization, hindering its transition into a widely accessible, standardized pharmaceutical product [81]. Additionally, personalized treatment necessitates phage susceptibility testing for each patient, which is time consuming, particularly in acute cases where the available phage bank may not cover the isolated pathogen [310]. While the European Pharmacopoeia has introduced flexible production standards for medicinal products used in phage therapy, and ongoing revisions to EU pharmaceutical legislation propose an “adaptive framework” for “variable composition phage medicinal products” [12, 311], significant challenges remain. These include the standardization of quality oversight for such a dynamic process, clear definition of therapeutic indications, and the implementation of effective postmarketing surveillance.

Phage therapy currently stands at a pivotal moment, transitioning from proof‐of‐concept toward clinical translation. With the integration of advanced tools like synthetic biology and artificial intelligence, precision phage therapy is expected to emerge. This will enable on‐demand design, rapid preparation, and on‐site application, offering a groundbreaking solution for infection prevention and control in the postantibiotic era. It is vital to note that the successful clinical translation of phage therapy depends not only on continuous technological advancements but also on the timely evolution of regulatory policies, sustained industry investment, and patient acceptance.

## Author Contributions

Jiajia Zheng, Ning Shen, and Huahao Fan designed the research. Zihe Zhou, Hanyu Fu, Mengzhe Li, Zhongyu Han, and Zhenchao Wu read and analyzed the manuscript. Zihe Zhou and Hanyu Fu wrote the manuscript. Jiajia Zheng, Ning Shen, Huahao Fan, Zihe Zhou, Hanyu Fu, and Mengzhe Li revised the manuscript. All authors contributed to the article and approved the submitted version.

## Conflicts of Interest

The authors declare no conflicts of interest.

## Ethics Statement

The authors have nothing to report.

## Data Availability

The authors have nothing to report.
